# No mafic layer in 80 km thick Tibetan crust

**DOI:** 10.1038/s41467-021-21420-z

**Published:** 2021-02-16

**Authors:** Gaochun Wang, Hans Thybo, Irina M. Artemieva

**Affiliations:** 1 State Key Laboratory of Lithospheric Evolution, Institute of Geology and Geophysics, Chinese Academy of Sciences, Beijing, China; 2 University of Chinese Academy of Sciences, Beijing, China; 3 Innovation Academy for Earth Science, CAS, Beijing, China; 4 Eurasia Institute of Earth Sciences, Istanbul Technical University, Istanbul, Turkey; 5 Center for Earth Evolution and Dynamics (CEED), University of Oslo, Oslo, Norway; 6 State Key Laboratory of Geological Processes and Mineral Resources, School of Earth Sciences, China University of Geosciences, Wuhan, China; 7 Department of Geophysics, Stanford University, Stanford, CA USA; 8 Marine Geodynamics Section, GEOMAR Helmholtz Center for Ocean Research, Kiel, Germany

**Keywords:** Geodynamics, Geophysics, Seismology

## Abstract

All models of the magmatic and plate tectonic processes that create continental crust predict the presence of a mafic lower crust. Earlier proposed crustal doubling in Tibet and the Himalayas by underthrusting of the Indian plate requires the presence of a mafic layer with high seismic P-wave velocity (*V*_*p*_ > 7.0 km/s) above the Moho. Our new seismic data demonstrates that some of the thickest crust on Earth in the middle Lhasa Terrane has exceptionally low velocity (*V*_*p*_ < 6.7 km/s) throughout the whole 80 km thick crust. Observed deep crustal earthquakes throughout the crustal column and thick lithosphere from seismic tomography imply low temperature crust. Therefore, the whole crust must consist of felsic rocks as any mafic layer would have high velocity unless the temperature of the crust were high. Our results form basis for alternative models for the formation of extremely thick juvenile crust with predominantly felsic composition in continental collision zones.

## Abstract (web summary)

Crustal doubling in Tibet and the Himalayas by underthrusting of the Indian plate is thought to require the presence of a mafic layer above the Moho. Here, the authors present seismic data which shows that the middle Lhasa Terrane has very low velocity (*V*_*p*_ < 6.7 km/s) throughout the 80 km thick crust, which they suggest is predominantly felsic in composition.

## Introduction

It is widely accepted that the continental lower crust is composed of mafic rocks^1^, as generally observed globally in the thick crust of orogens, shields, and platforms^2–8^. It has been suggested that the lower crust does not need to be basaltic^9^, but until now all seismic observations show high P-wave velocity, which requires that the bulk composition of the lower crust must include at least 20–40% of mafic rocks^10^. The ongoing Indo-Asian continental collision has created some of the thickest crust on Earth, which is conventionally assumed to include a thick mafic lower crust with high seismic velocity (*V*_*p*_ > 7.0 km/s)^11–17^, although the lowest crustal velocity remains relatively unconstrained by the hitherto available data.

The crustal velocity structure of inner Tibet is sparsely constrained due to complicated fieldwork logistics under the extreme climatic and topographic conditions. Tectonically, the Himalayan-Tibetan orogen includes a series of amalgamated terranes, which are separated by major suture zones. The Tethyan Himalaya (TH) has Gondwana affinity and may have formed part of the Indian Plate. Further north, the Lhasa terrane (LT) is part of the Eurasian Plate, and it is usually assumed to be underthrust by the Indian Plate. The resulting crustal doubling^11–17^ implies that the crust should contain large amounts of mafic material.

**Fig. 1:**
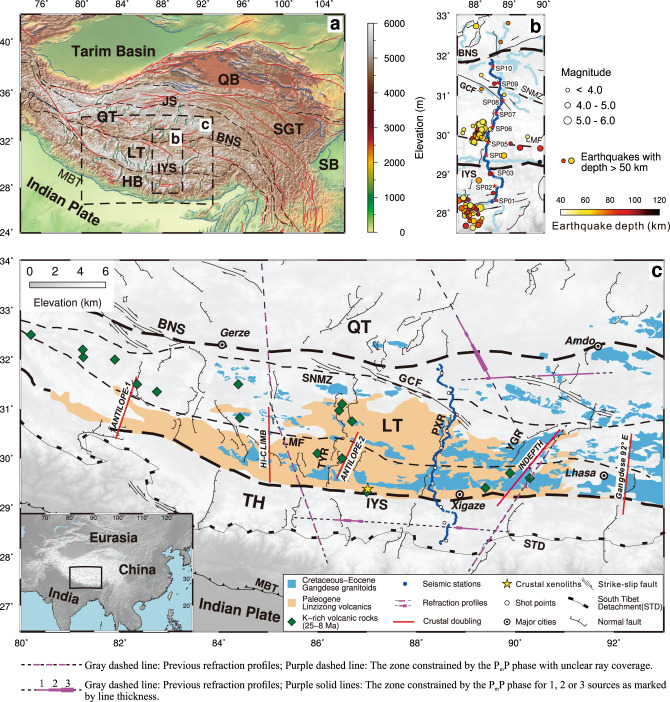
Topographic map, seismic profile location in Tibet and geologic setting. **a** Topographic map with major tectonic units and sutures. Black rectangles show locations of maps in **b** and **c**. **b**. Location of seismic refraction/wide-angle reflection profile across LT and TH. Blue dots: seismometer locations; red stars: seismic source locations; circles: location and hypocentre depth for all deep (>50 km depth) earthquakes within the map from 1990 to 2017 (ISC, http://www.isc.ac.uk/). **c** Major geological structures^62,63^ and areal coverage of three series of volcanism^48–50,64^. Locations of proposed underthrusted Indian Plate from receiver functions (“crustal doubling”) are marked^12,13,15–17^. Earlier seismic refraction/wide-angle reflection profiles are shown by stippled lines, and thick purple lines indicate ray coverage for Moho reflections. Insert shows location of study area. QB Qaidam Basin, SB Sichuan Basin, SGT Songpan-Ganzi Terrane, QT Qiangtang Terrane, LT Lhasa Terrane, HB Himalayan Block, TH Tethyan Himalaya, BN Bangong-Nujiang suture, GCF Gyaring Co fault, JS Jinshajiang suture, LMF Luobadui–Milashan Fault, IYS Indus–Yarlung suture, STD South Tibet Detachment, MBT Main boundary thrust, SNMZ Shiquan River–Nam Tso Mélange Zone, TYR Tangra YumCo rift, PXR Pumqu-Xianza rift, YGR Yadong-Gulu rift.

Most of the few available controlled source seismic profiles in the LT (Fig. 1c) were acquired at low resolution and with limited depth coverage due to sparse horizontal sampling^18^. These relatively old profiles, acquired between 1974 and 1992, provide indication for low velocity and felsic material in the crust in LT down to the Moho at ca. 60 km depth^18–20^. All seismic models of thicker crust sampled in Tibet and elsewhere on Earth, including the Andes, include a high-velocity lower crust^18,21–24^. In particular, models close to the suture between the Lhasa and Qiangtang terranes in northern LT include a lower crust with high velocity and an unconstrained high velocity gradient from 6.6 to 7.3 km/s between 42 and 62 km depth around 89–92 °E^21,22^ and up-to 6.9–7.3 km/s between 50 and 75 km depth around 93 °E^22^. However, due to sparse distribution of shot points, these results have large uncertainty and the velocity gradient is unconstrained by the data in both profiles.

Surface wave tomography results include a low shear wave velocity feature in the crust at the depth range of 20–40 km, which is interpreted as partial melting or mineral alignment in most of central Tibet^25–27^. The reference S-wave velocity model for China^26^ includes low velocity down to Moho at 60–70 km depth in most of southern Tibet, but there is a trade-off between the velocity of the lower crust and the upper mantle, such that the lower crustal velocity determined from surface wave tomography has relatively large uncertainty. Joint inversion of surface waves and receiver functions has identified an up-to 75 km thick crust in western Tibet, including a high-velocity lower crust^28^.

Magnetotelluric data identify low resistivity^29^ in the middle crust along the southern margin of the Tibetan plateau and in northern and eastern Tibet^30^, which the authors explain by the presence of minor conducting phases, melt, or water^25,31^, consistent with low seismic velocity in the middle crust^25,26^. However, the models indicate that the resistivity increases in the lower crust below ~50 km depth in the LT. The high resistivity is interpreted as a relatively cold and thick lithospheric structure compared to the Qiangtang terrane, and it may also indicate high seismic wave velocity^32^.

Receiver function results image two lower crustal converters in parts of the LT, which have been interpreted as indication for crustal doubling (Fig. 1c) caused by the presence of subducted Indian lower crust below southern Tibet^11–17^. This model implies that a mafic crustal layer, possibly in eclogite facies with high seismic velocity^13^, is present below Tibet from the TH in the south to the Qiangtang Terrane in the north^11^ or at least to 31 °N in northern LT^13,14^. Receiver function studies report Moho depths of up-to ~70–75 km in the LT^11–14^ and exceptionally thick crust extending to depths of around 90 km in the Quintang Block in western Tibet^33^ and in Pamir^34^. These interpretations assume that the lower crust has high seismic velocities which, however, are unconstrained by the applied methods. Therefore, the interpretations of the crustal lithology, the nature of the seismic converters, and their depths so far remain unconstrained.

Here, we present and interpret the velocity structure in the TH and the LT in central-southern Tibet along a NS-striking wide-angle reflection and refraction profile. Comparison of our results with typical crustal lithologies shows that the whole crust consists of felsic rocks, including the lower crust at depths of ~50–80 km. The presence of felsic lower crustal rocks to depths of 80 km in some of the thickest crust on Earth provides data-based evidence for new understanding of the evolution of the continental crust and the formation of the Tibetan plateau.

## Results

### Seismic data and interpretation

Our new 450 km long, NS-striking controlled source seismic profile (Fig. 1) constrains the velocity structure of the whole crust at high resolution in the LT and TH. This refraction/wide-angle reflection seismic profile extends at around 88.5 °E between ca. 28.3 °N in the TH and the Bangong-Nujiang suture (BNS) at ca. 32 °N with a station spacing of ~1.5 km and a nominal shot spacing of ~45 km (Fig. 1b). In total, 311 short-period geophones recorded signals from ten large shots, each with 2000–4000 kg of explosives detonated in ~50 m deep boreholes (Fig. 1b, “Methods” section).

The data were interpreted by phase correlation and traveltime picking of the main seismic phases from the crust and uppermost mantle, followed by tomographic inversion and ray tracing traveltime modeling of the crustal velocity structure as well as extensive tests of uncertainties in the model (“Methods” section, Supplementary Figs. 1, 5, 6 and 7).

### Seismic model

**Fig. 2:**
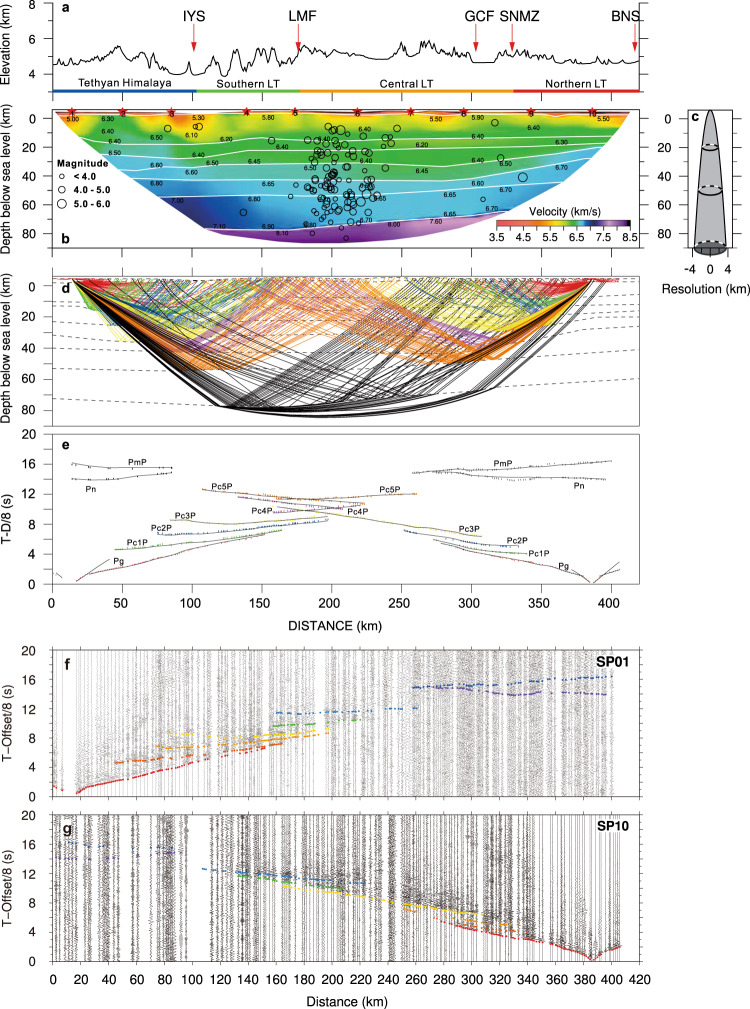
Seismic P-wave velocity structure along our wide-angle seismic profile across LT and TH. **a** Elevation of the seismic profile with location of faults and suture zones (red arrows) and location of different terranes (color line). **b** Crustal velocity structure along the seismic profile. Seismic sources: red stars; velocity discontinuities: white lines; location of earthquakes within a 100 km wide corridor: black circles; **c** Horizontal resolution for the velocity model estimated as the Fresnel zone width. **d** Ray tracing coverage of the seismic model for the end shots 01 and 10, illustrating the high resolution of the lower crust and depth to Moho, see also Supplementary Fig. 5. **e** Traveltime fit for seismic phases for the two end shots. Lines show calculated traveltimes and vertical bars show observed traveltimes with length of bar corresponding to uncertainty of pick. **f** Seismic section for SP01 reduced by 8 km/s with traveltime picks. **g** Seismic section for the reversed SP10 with traveltime picks. Abbreviations as in Fig. 1.

**Fig. 3:**
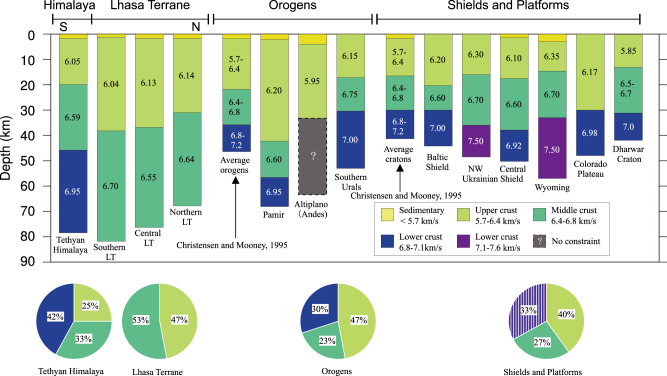
Vertical crustal seismic compressional velocity sections. Velocity sections for different parts of the study area shown together with vertical models from other regions with thick crust (Moho depth >45 km) and the Dharwar Craton in India^7^, as well as the global average crustal structure^8^ of orogens and cratons. LT Lhasa Terrane, NW Northern-western. Only the velocity sections for the Lhasa Terrane lack a high-velocity lower crust corresponding to a mafic composition, which makes its structure unique.

Our seismic velocity model (Fig. 2) challenges conventional models of continental crustal structure and previous models of the Tibetan region. It shows the following characteristics:

1. Presence of very thick crust in LT extending down to 80 ± 2 km, which is among the thickest crust observed on Earth and thicker than generally observed in LT.
2. Distinct difference in velocity structure between the crust of LT and TH, which challenges previous interpretations based on lower-resolution seismic data.
3. Presence of a high-velocity zone in the lower crust of TH with *V*_*p*_ > 7.0 ± 0.2 km/s.
4. Absence of a high velocity lower crust in LT in contrast to any other thick continental crust, which conventionally includes a thick mafic lower crustal layer (Fig. 3). The extremely low velocity in the whole crust down to Moho at 80 ± 2 km in LT is everywhere <6.7 ± 0.2 km/s (on average 6.32–6.45 km/s for the whole crust), whereas velocities >7.0 km/s are everywhere observed in other extremely thick crust.
5. Presence of a continuous Moho along the whole seismic profile, which indicates that the Indian lower crust cannot subduct into the upper mantle below TH and LT.
6. Normal Pn velocity (8.1 ± 0.2 km/s) from TH to the middle part of LT and low Pn velocity (7.6 ± 0.2 km/s) further to the north.

The presence of a high-velocity lower crust in the southern end of the profile is consistent with underthrusting of Indian Plate lower crust below TH and at maximum ca. 50 km into LT (Fig. 2). The continuous and smooth Moho interface and constant Pn velocity show that the lower crust of the Indian Plate cannot extend further north than to 30 °N. The observed high-velocity lower crust below TH is characteristic of the Indian Plate lower crust, e.g., the crust of the Dharwar Craton in India includes a lower crustal layer between 32 and 42 km depth with a velocity >7.0 km/s^7,35^ (Fig. 3), whereas the LT lower crustal velocity is small (<6.7 km/s). This shows that the Indian crust cannot underthrust LT because the lower crustal velocity then would be high. If the Indian crust were subducting into the upper mantle below LT, the Moho would be disrupted and low Pn velocity would be observed in the sub-Moho mantle (Fig. 2b). The present well constrained seismic observation contradicts earlier suggestions that Indian crust extends across LT and dips into the upper mantle^11–15^.

The unusual low-velocity continental crust in LT is well constrained by our newly acquired, high-resolution seismic data. The resolution of depth and velocity are ±2 km and ±0.2 km/s, respectively (Supplementary Fig. 1, “Methods” section). The horizontal resolution varies with depth and is better than 8 km at the Moho at ~80 km depth (Fig. 2c). We have carried out extensive robustness tests based on linear least-squares inversion, which demonstrate that neither higher lower crustal velocity nor deeper Moho can explain the seismic observations (Supplementary Figs. 6 and 7, “Methods” section).

### Interpretation of lithology

**Fig. 4:**
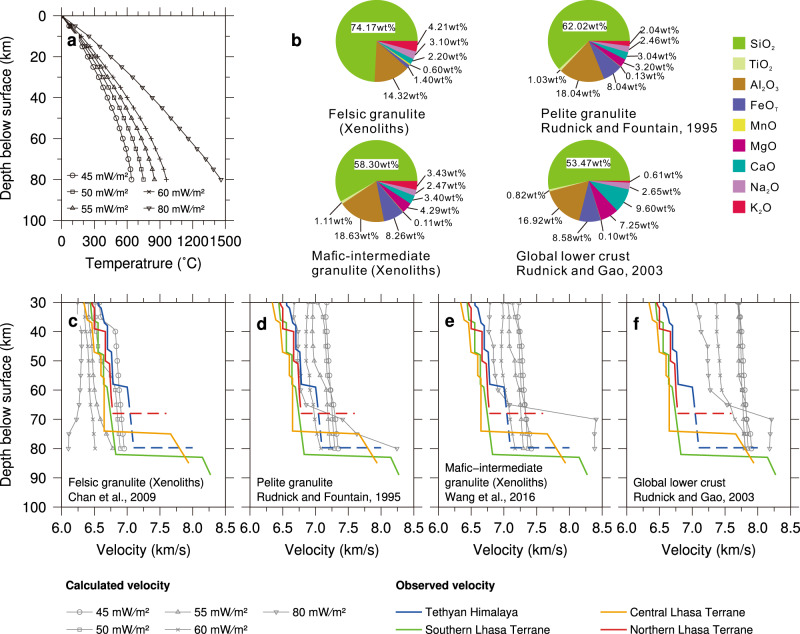
Observed vertical velocity profiles compared with calculated velocities for major relevant rock types at different temperature. Calculations were carried out with the Perple_X_6.8.5 phase equilibrium modeling program^36^ for the internally consistent thermodynamic dataset ds62^37^. The composition of rocks is chosen according to xenoliths (felsic granulite and mafic-intermediate granulite) in southern Tibet^39,40^ as well as a pelite granulite^1^ and a global average lower crustal model^41^. **a** Calculated geotherms for different assumed heat flow values. **b** Composition of the four chosen rock types. **c**–**f** Observed vertical velocity profiles in the four characteristic tectonic parts along the profile, superimposed by calculated velocity profiles for the four selected compositions.

To interpret the possible composition of the anomalous crust of LT, we compare crustal velocity-depth profiles for TH and southern, central and northern LT with velocity-depth profiles for typical crustal lithologies (Fig. 4), calculated by the thermodynamic, phase equilibrium code, Perple_X^36,37^, for relevant temperature regimes^38^. The chosen compositions represent felsic and mafic-to-intermediate granulites as observed in xenoliths from southern LT (~29.3 ^o^N, 87 ^o^E) ca. 30 km north of the IYS^39,40^ as well as pelite granulite^1^ and a global average continental lower crust^41^.

No reliable heat flow measurements exist in the study region because all measurements were carried out in shallow boreholes along roads that follow fault zones and include hot springs in their vicinity^42,43^. However, the presence of deep earthquakes in LT (Figs. 1b and 2b), down to the Moho at ~80 km depth, demonstrates that the crustal temperatures are low (<550–600 °C), because otherwise the crust would not be able to sustain sufficient stress to generate earthquakes^44^. Low heat flow has also been inferred from integrated geophysical-petrological interpretation^32^, and is supported by the finding of up-to 300 km thick lithosphere from seismic tomography in the same area^45,46^. In this low temperature regime only felsic rock types, such as felsic granulite, can explain the observed low *Vp* velocities in the lower part of this exceptionally thick crust (Fig. 4c). Even at very high temperature, mafic composition cannot explain the low *Vp* at the base of the LT crust (Fig. 4d–f).

The observed seismic velocities in the lower crust of TH are consistent with granulite compositions if temperatures are relatively high (800–900 °C at Moho) or relatively felsic compositions if temperatures are similar to LT (Fig. 4d, e). The calculated average global lower crustal composition (Fig. 4f) predicts seismic velocities that are too high to explain the observed velocities for any temperature regime.

## Discussion

We conclude that the whole crust in LT along our profile is felsic from surface to the Moho, despite it represents some of the thickest crust on Earth with a thickness of 80 km. This precludes the presence of mafic lower crust and therefore the lower crust of LT cannot consist of underthrust crust of the Indian Plate along our profile, contrary to earlier models based on receiver function interpretations^11–15^. It also precludes the presence of a lower crustal layer in eclogite facies above the Moho^13,14^ along the profile, because such rocks would have high seismic velocity. The model shows that the Indian plate cannot underthrust the LT for more than ca. 50 km in the region around our profile at ca. 88.5 °E, whereas the underthrusting may extend for 100–150 km north of the IYS in a receiver function profile at around 85 °E^13^.

The concentration of deep (down to 80 km depth) seismicity in the felsic low-velocity crust of central LT is remarkable. Its origin should be tectonic, because earthquakes associated with metamorphic transformations into eclogite facies, as recently proposed for TH^47^, cannot be generated in LT due to the lack of mafic material.

The crustal structure of the LT (thickness, velocity and composition) is different from crust observed anywhere else on Earth, including other regions with exceptionally thick crust (Fig. 3), which challenges classical views on the nature of the continental crust. We stress that volcanic rocks from the LT (Fig. 1c) are characteristically almost entirely felsic^48,49^ in contrast to the surrounding terranes^49–51^. We speculate that this surprising crustal structure of LT indicates that it formed by a new type of tectono-magmatic process.

If the 80 km thick felsic crust formed by magmatic processes, there must have been equally massive production of mafic rocks that are no longer present in LT. This implies massive removal of the mafic part of the new crust, which may have contributed to the high topography in Tibet^52^. However, it appears unlikely that new crust formed by magmatic processes in a subduction environment at any time could include an 80 km thick felsic crust above a similarly thick mafic lower crust. Such extremely thick crust would be unstable and metamorphic processes in the lower, mafic crust would soon form rocks with very high density. We therefore speculate if either tectonic processes repeatedly separated the newly formed upper felsic crust from the lower mafic crust, or if the newly formed mafic lower crust episodically delaminated due to metamorphic reactions, while the crust-forming magmatic processes were active. The presence of significant amounts of water from the long-lasting subduction of the Tethyan oceans^48^ and high pressure in superdeep crustal layers would facilitate metamorphic reactions. The negative buoyancy of eclogite facies rocks with density substantially higher than mantle peridotites would make the newly formed deep lower crust prone to recycling into the mantle each time the eclogitic portion reached a critical mass, leaving behind the extremely thick, entirely felsic crust of LT. This mechanism has similarity to a recently proposed crust-forming process^10,53^, although the exceptionally thick crust may be essential for the process to operate efficiently.

Seismic Pn velocity of the sub-Moho mantle supports this model. Whereas the Pn velocity of 8.1 km/s along most of the profile is consistent with a peridotitic mantle, the Pn velocity is unusually low (7.6 km/s) in northern LT below a clear Moho reflector despite the crustal structure does not change across the LT. This observation is consistent with recent results of Pn tomography of Tibet^54^. The low Pn velocity requires either the presence of partial melt and high temperature in mantle material or the presence of crustal material below the seismic Moho, such as mafic granulite partially in eclogite facies. For an eclogite velocity of 8.6 km/s and a typical lower crustal velocity of 7.2 km/s, the Pn velocity of 7.6 km/s implies that ca. 30% of the mafic granulite is in eclogite facies below the Moho. Assuming densities of 3.0 and 3.6 g/cm^3^ for mafic granulite and eclogite, the sub-Moho density is ca. 3.2 g/cm^3^, which is less than mantle peridotite density. We, therefore, speculate that the metamorphic process in northern LT did not fully transform the mafic part of the newly formed crust into eclogite facies, and that this part never delaminated due to insufficient density and volume so that the low Pn velocity in northern LT has a compositional instead of thermal origin.

The elevation of the LT was ~4 km at ~60 Ma after which uplift has been slow and with small amplitude until now^55,56^. If the crust-forming processes involved continuous or episodic loss of lower crustal material, the uplift of the Tibetan plateau was probably not abrupt due to sudden loss of the whole high-density lower crust^52^. Instead the rise of LT would have been continuous and gradual, simultaneous with the crust-forming processes, and the rate of uplift may provide a proxy for the rate of felsic crust production.

The absence of a mafic lower crust in the central part of the middle LT precludes future delamination due to lack of mafic rocks that may transform into high density eclogitic rocks. Therefore, models proposing future destruction of the entire high-altitude Tibetan plateau by crustal delamination, such as proposed for western Europe^52^, followed by isostatic topographic collapse^57^, may be seriously questioned for LT. With the present crustal structure, destruction of the Tibetan plateau in LT can only happen by surface erosional processes or by basal thermal erosion.

Our results provide the first direct geophysical observation of exceptionally thick, fully felsic crust, as determined from unexpected low seismic velocity and low temperature for the 80 km thick crust in the middle part of the LT. This finding challenges earlier proposed geodynamic models for Tibet. Our results demonstrate that some of the thickest crust on Earth lacks a mafic lower layer and, therefore, its presence is inconsistent with “classic” crust-forming models.

## Methods

### Seismic data

Our ~450 km long refraction/wide-angle reflection seismic profile was acquired along ~88.5 °E longitude from September to October 2016 between ca. 28.3 °N in the TH and the BNS at ca. 32 °N (Fig. 1b). Instruments were deployed close to the only existing road in the area, which follows a major fault-controlled depression in the surface, the Pumqu-Xianza rift. The seismic profile includes data from 311 seismic stations and ten shots (Fig. 1b) with a station spacing of ~1.5 km and a nominal shot spacing of ~45 km. The seismic instruments were equipped with 2.5 Hz short-period 3D component geophones and the sample rate was 10 ms. The seismic sources were four shots each with 4000 kg of explosives each (shots SP01, SP02, SP09, and SP10) at the ends of the profile and six shots each with 2000 kg of explosives (shots SP03–SP08, Fig. 1b and Supplementary Table 1). All shots included charges in several boreholes at a depth of ~50 m.

### Seismic analysis

Interpretation of the data includes phase correlation and traveltime picking of the main seismic phases from the crust and uppermost mantle (Fig. 2 and Supplementary Figs. 2–4), followed by tomographic inversion and ray tracing traveltime modeling of the crustal velocity structure. The resolution of depth and velocity are ±2 km and ±0.2 km/s, respectively (Supplementary Fig. 1), and the horizontal resolution varies with depth as illustrated in Fig. 2c.

The seismic phases from the crust and upper mantle were correlated and traveltimes were picked by use of the ZPlot software package (modified by P. Środa from ZPlot written by C. A. Zelt^58^, Supplementary Fig. 2). The traveltime tomography and ray tracing (Fig. 2 and Supplementary Figs. 2 and 5) to obtain the P-wave velocity model were carried out with the software packages FAST^59^ and rayinvr^58^. The ray tracing modeling included a state-of-the-art, top-to-bottom modeling procedure. We took into consideration the traveltime characteristics of refracted and reflected phases in the crust and uppermost mantle. This includes matching the apparent velocities of refracted phases, ensuring match of reciprocal arrivals, to obtain true velocities in the Earth. Because the deeper seismic phases primarily consist of overcritical reflections, we paid specific attention to match traveltimes both around the critical point and in the far field reflections, which primarily constrain the true velocities in the Earth, as well as the offset of the critical point for the reflections, which constrains the velocity contrast across the reflectors. The model is well constrained by reversed arrivals, also for the Pn phase from the sub-Moho mantle.

### Resolution of the seismic model

We use the diagonal values of the resolution matrix to test the model reliability for depths and velocities^60^. A parameter of the velocity model is considered reliable if its diagonal value is larger than 0.5 within a depth variation of Δd = ±2 km. Almost all nodes within the area covered by seismic rays are resolvable within 2 km at all depths (Supplementary Fig. 1a). By adding an alternating velocity perturbation of ±0.2 km/s to the velocity nodes followed by inversion for the crustal velocity structure, we obtained the diagonal values of the resolution matrix for velocities (Supplementary Fig. 1b). These values are larger than 0.5 in almost the whole central part of the seismic profile, where the ray coverage is denser than at the ends of the profile (Supplementary Fig. 5). High resolution is also found around the IYS zone, although a few nodes have values between 0.25 and 0.50.

### Traveltime fit for seismic phases

Our velocity model has high resolution in the shallow crust. The P waves traveling in the upper crystalline crust (Pg phase) can be traced to offsets of more than 100 km and are identified in all the seismic sections with clear onsets, which enables precise determination of arrival times. In the following description we disregard the data from the low-energy shot SP02, which only carried observable energy to ca. 60 km, and only for the Pg phase. The Pg phase can be picked out to between ~40 and 110 km offset in all other seismic sections (Supplementary Fig. 2). They show a clear delay across the IYS, which indicates that our velocity model also constrains the low velocity structure beneath the IYS. The sections show higher signal-to-noise level in LT than in TH, which indicates that the seismic signals propagate better from TH into LT than in the opposite direction and that the noise level may be higher in TH than in LT. These observations are similar to observations of better transmission from sedimentary basins to cratonic crust than in the opposite direction^61^. However, the signals in all sections are confidently correlated and the traveltimes are checked for reciprocity.

We picked five different types of reflection arrivals (Pc1P, Pc2P, Pc3P, Pc4P, and Pc5P) in the crust from the interfaces C1, C2, C3, C4, and C5 as well as the PmP reflection from the Moho and the Pn phase, which is the refracted wave below the Moho (Supplementary Figs. 2 and 3). All the reflection arrivals are clear and readily recognizable, although they have a relatively lower signal-to-noise ratio than the P_g_ phase. We estimate the crustal velocities deeper than ca. 10 km from the slopes of the reflections, assuming that the observed reflections are super-critical, such that the slope at the onset (critical point) represents the apparent velocity below each discontinuity, and the slope at the far end represents the maximum apparent velocity immediately above the discontinuity, corresponding to the merge with the deepest diving wave in the layer. We further used the observed critical distances for the reflections for determination of the velocity contrast across the discontinuities. The apparent velocity is also affected by the dip of the discontinuities, which generally is small, and which we correct for by the modeling and inversion. The velocity in the uppermost mantle in the central part of the section is well constrained by reversed refracted Pn arrivals.

The Pc1P phase is the reflection phase from interface C1 at depths of ~15–21 km. It generally appears at 20–80 km offset and can be traced to ~150 km offset. This phase has high signal-to-noise ratio in the sections for shots 01, 03, 04, 05, 06, 07, and 08 and is observable in the sections for shots 09 and 10. Pc1P merges with the Pg phase at far offsets, which provides credibility to our approach for estimating the deeper velocities in the model from the slopes of reflected phases. The slopes for the Pc1P phase at critical points (apparent velocity below interface C1) range from about 6.4 to 6.7 km/s and the slopes at far ends (apparent velocity above the interface C1) range from about 6.2 to 6.5 km/s, with the smallest values around IYS.

The Pc2P phase, from interface C2 at depths of ~22–33 km, has apparent velocities in the interval of 6.3–6.7 km/s, with about 6.7 km/s at critical points and about 6.3–6.5 km/s at far ends. The Pc2P phase has large amplitude in the seismic sections of shots 05, 06, 07, 08, and 09, in particular between 280 and 380 km along the profile for shot SP05 and 300–380 km for shot SP06. The apparent velocity of 6.3–6.5 km/s above the C2-reflector is slower than for the Pc1P phase in the central to northern part of the profile, where it indicates the presence of a possible low-velocity zone between interfaces C1 and C2.

The Pc3P reflection with an apparent velocity of 6.4–6.5 km/s from interface C3 at depths of ~27–42 km is slightly weaker than Pc2P. It is identified in all seismic sections except for shot SP02, and it shows strong amplitudes relative to other phases at the far end. The slopes for Pc3P phase are about 6.75 km/s at critical points and about 6.5 at far ends.

The Pc4P from interface C4 at depths of 33–48 depth is traced over long offset intervals in the sections for shots 01, 03, and 10 and over relatively shorter intervals in the other seismic sections. The slopes for Pc4P phase are about 6.7–7.0 km/s at critical points and 6.5–6.7 km/s at far ends.

The Pc5P at depths of 47–59 km with strong amplitude and good continuity can confidently be traced from 150 to 250 km offset in the section for shot SP01 and from 165 to 280 km offset in the section for shot SP10, whereas it only appears at the end of the profile in other sections.

The ray tracing results show that each phase provides good spatial ray coverage at all depths in the velocity model because the seismic phases generally are correlated over large offset intervals (Supplementary Fig. 2). The crustal velocity structure is therefore well constrained as is also evident from the resolution test (Supplementary Fig. 1).

The PmP phases are correlated in the seismic sections for shots 01, 03, 04, 05, 08, 09, and 10 with variable waveform and amplitude. For shot SP01 (Supplementary Figs. 2a and 4a), the PmP reflection is observed from 250 to 360 km offset with weak amplitude. The PmP is strong for shot SP03 (Supplementary Figs. 2c and 4b) between 200 and 310 km offset, i.e., out to the end of the profile. For the central shot points, the Moho reflection is only identified at the far ends of the profiles for shots SP04, SP05, as well as for shots SP08 and SP10 (Supplementary Figs. 2d, e, h, j and 4c, e), and over the offset interval 220–300 km for shot point SP09 (Supplementary Figs. 2i and 4d). The far offset amplitude of the PmP reflection is strong for SP8 and SP10 (Supplementary Fig. 4c, e), probably because the reflection is observed in the down-dip direction, which by reciprocity improves the reliability of the PmP observations for SP1 and SP3 (Supplementary Fig. 4a, b). As a result, the lower crustal velocities and Moho depths in our velocity model are well constrained between 110 and 370 km along the profile with a small gap between 310 and 330 km (Supplementary Fig. 2d, e).

To further test the velocity resolution, we also calculate the arrival times of the PmP phases with different velocities in the lower crust (Supplementary Fig. 6). The top lines in each slice show the calculated arrivals with the velocity of 6.5 km/s in the lower crust of LT, and the bottom lines show the calculated arrivals with the velocity of 6.8 km/s in the lower crust of LT. The shadow zones between the top and bottom lines show the arrival times between the velocity of 6.5 and 6.8 km/s. This test clearly illustrates that the velocity in the lower crust cannot be larger than 6.7 km/s.

The Pn phases are correlated in the seismic sections of shots 01, 03, 08, 09, and 10. Clear arrivals for this diving wave in the upper mantle are identified from 250 to 390 km offset for shot SP01 (Supplementary Figs. 2a and 3a), although the phase is not identified between 335 and 350 km offset. For shot SP03 (Supplementary Figs. 2c and 3b), the Pn phase is very clear from 300 to 330 km offset; there are clear arrivals from 250 to 290 km offset in the seismic section of shot SP08 (Supplementary Figs. 2h and 3c); but the Pn phases can only be identified on some of the seismograms from 280 to 320 km offset in the seismic sections of shots SP09 and SP10 (Supplementary Figs. 2i, j and 3d, e). The Pn phases provide good ray coverage in the uppermost mantle between 120 and 320 km along the profile.

The 2-D ray tracing velocity model in Fig. 2b is constrained by about 99.5% of the traveltimes picked from the seismic section (Supplementary Table 2). A picking error of 100 ms is associated with the Ps and Pg phase, i.e., the first arrival refractions from the sedimentary cover and the shallow crystalline basement, and an uncertainty of 150 ms is associated with the traveltime picks for intra-crustal reflections, PmP and the Pn. With these estimated uncertainties, the *χ*^2^ measure for the calculated traveltimes is ~2.6 for the Pg phase and smaller for other phases, resulting in an overall *χ*^2^ of 1.7 for all phases constraining the velocity model (Supplementary Table 2). A perfectly fitting model within uncertainties would result in a *χ*^2^ of 1. The rms traveltime residual (trms) is less than the associated picking error for all phases with an average value of 104 ms. All the calculated traveltimes from each shot match the traveltime picks with few outliers as indicated by the global *χ*^2^ measure.

The plot of ray coverage (Supplementary Fig. 5) and the traveltime residuals (Supplementary Table 2) show that the crustal velocity model is well constrained by the interpreted seismic phases.

### Test of the robustness of lower crustal velocity and Moho depth

The exceptionally low velocity of the 80 km deep lower crust in our seismic model is a main finding. Resolution test shows that the model is very well constrained, and the PmP and Pn phases are modeled within a rms traveltime misfit of 99 ms (Supplementary Table 2 and Supplementary Fig. 2). Here, we provide further test of the robustness of this key observation (Supplementary Fig. 7).

We test if the lower crustal velocity may be higher than in our preferred model by manually changing it to a fixed value of 7.0 km/s and then inverting for Moho depth. The result shows that there is no acceptable solution for this high lower crustal velocity, and that the traveltime misfit remains larger than 220 ms for all models (Supplementary Fig. 7i), which is larger than the estimated uncertainty of the picks. Allowing the inversion algorithm to invert simultaneously for lower crustal velocity and Moho depth leads to a model similar to our preferred model with a lower crustal velocity of 6.7 km/s and a Moho depth of ca. 80 km in central LT (Supplementary Fig. 7ii).

We further test if the Moho may be deeper than in our preferred model by manually fixing all Moho depths in the model to 5 km deeper than in the preferred model and then inverting for the lower crustal velocity. The result shows that there is no acceptable solution and that the best obtainable model has a rms traveltime misfit of 225 ms and that this model does not explain 19 out of 279 traveltime picks (Supplementary Fig. 7iii). Allowing the inversion algorithm to invert simultaneously for lower crustal velocity and Moho depth leads to a model similar to our preferred model with a lower crustal velocity of 6.7 km/s and a slightly shallower Moho than in the preferred model, although with larger rms traveltime misfit of 115 ms and loss of coverage for 20 out of 279 traveltime picks (Supplementary Fig. 7iv).

## Supplementary information

Supplementary Information

## Data Availability

All data are available in the manuscript and in Supplementary Figs. 2 and 3.

## Code availability

All applied computer code is available from the author, Dr. Colin Zelt at his web-site: https://terra.rice.edu/department/faculty/zelt/.
