# Supplementary material for: No mafic layer in 80 km thick Tibetan crust
Source: Nat Commun. 2021 Feb 16;12:1069. doi: 10.1038/s41467-021-21420-z (PMC7886915; doi:10.1038/s41467-021-21420-z)
Supplement: Supplementary file 1 — Supplementary Information [file 41467_2021_21420_MOESM1_ESM.pdf]

Supplementary Information for

No mafic layer in 80 km thick Tibetan crust

Gaochun Wang, Hans Thybo, Irina M. Artemieva

Correspondence to: thybo@geo.uio.no

This PDF file includes:

Supplementary Figure 1. Resolution test for the velocity model.
Supplementary Figure 2. Seismic sections with reduction velocity 6 km/s for all ten shots.
Supplementary Figure 3: Seismic sections with reduction velocity 8 km/s for shot points 1, 3, 8, 9 and10.
Supplementary Figure 4. Seismic sections with reduction velocity 6.7 km/s for shot points 1, 3, 8, 9 and 10.
Supplementary Figure 5. Ray tracing results for the seismic phases.
Supplementary Figure 6. Test of lower crustal velocity.
Supplementary Figure 7. Robustness test of lower crustal velocity and Moho depth.
Supplementary Table 1. Shot Parameters for the data acquisition.
Supplementary Table 2. Statistical parameters of the seismic modelling for different picks.

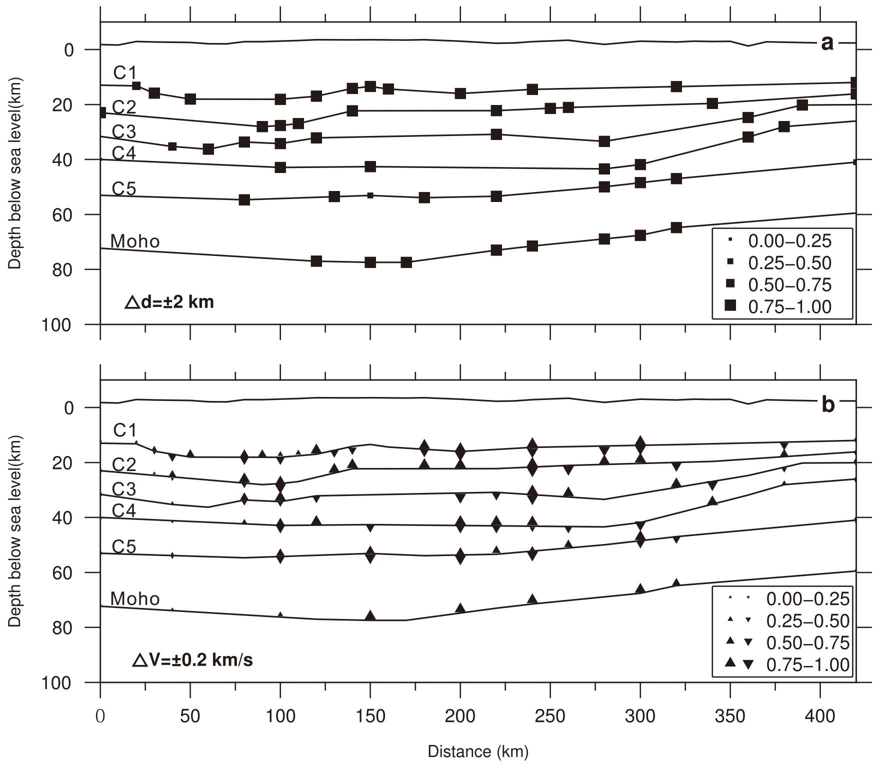

Supplementary Figure 1. Resolution test for the velocity model. The diagonal values of the resolution matrix were calculated to test the model reliability for depths and velocities. a. The depth reliability ($\Delta d = \pm 2$ km) for the node depths. b. The velocity reliability ($\Delta V = \pm 0.2$ km/s) for the node velocities. The interfaces in the crust are marked by C1, C2, C3, C4 and C5.

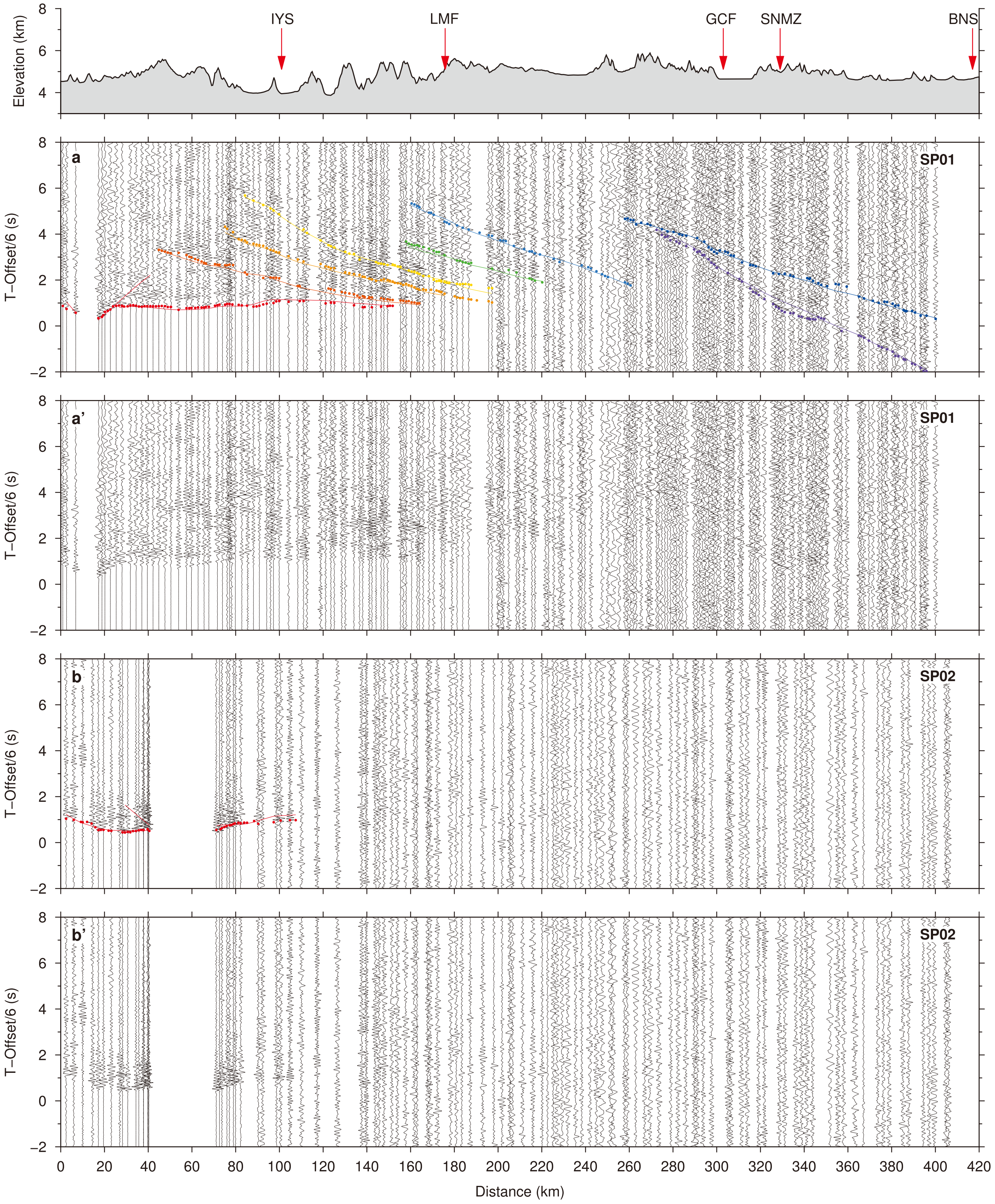

Supplementary Figure 2. Seismic sections for the ten shots. The seismic sections include the picks (dots) and the traveltimes (solid lines) calculated for the final velocity model. Topographic profile with major tectonic features is inserted on top of each set of seismic sections. The travel time is reduced by a velocity of 6.0 km/s. Abbreviations: IYS: Indus–Yarlung suture, LMF: Luobadui–Milashan fault, GCF: Gyaring Co fault, SNMZ: Shiquan River–Nam Tso Mélange Zone, BNS: Bangong-Nujiang suture.

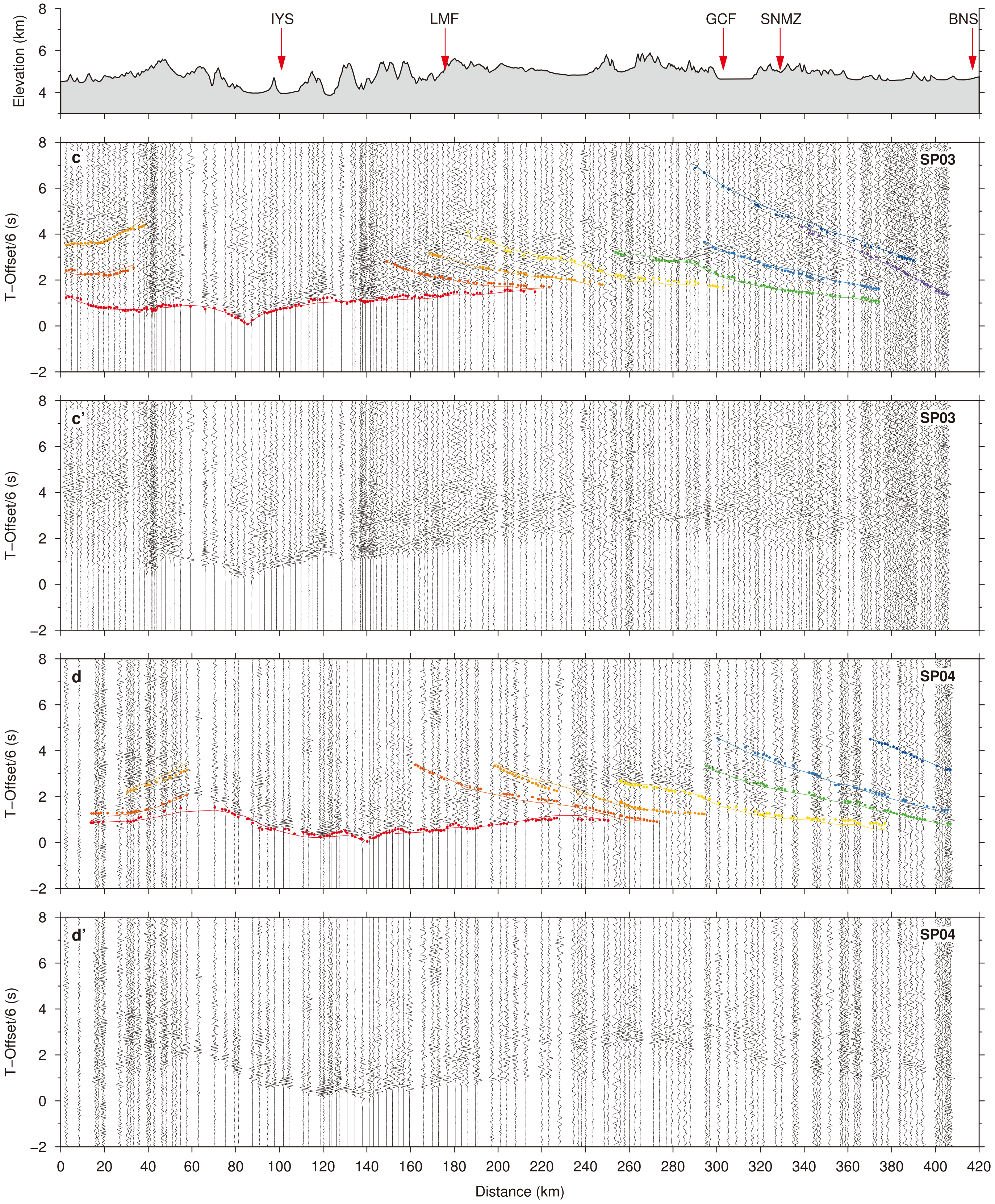

Supplementary Figure 2. Seismic sections for the ten shots (continued).

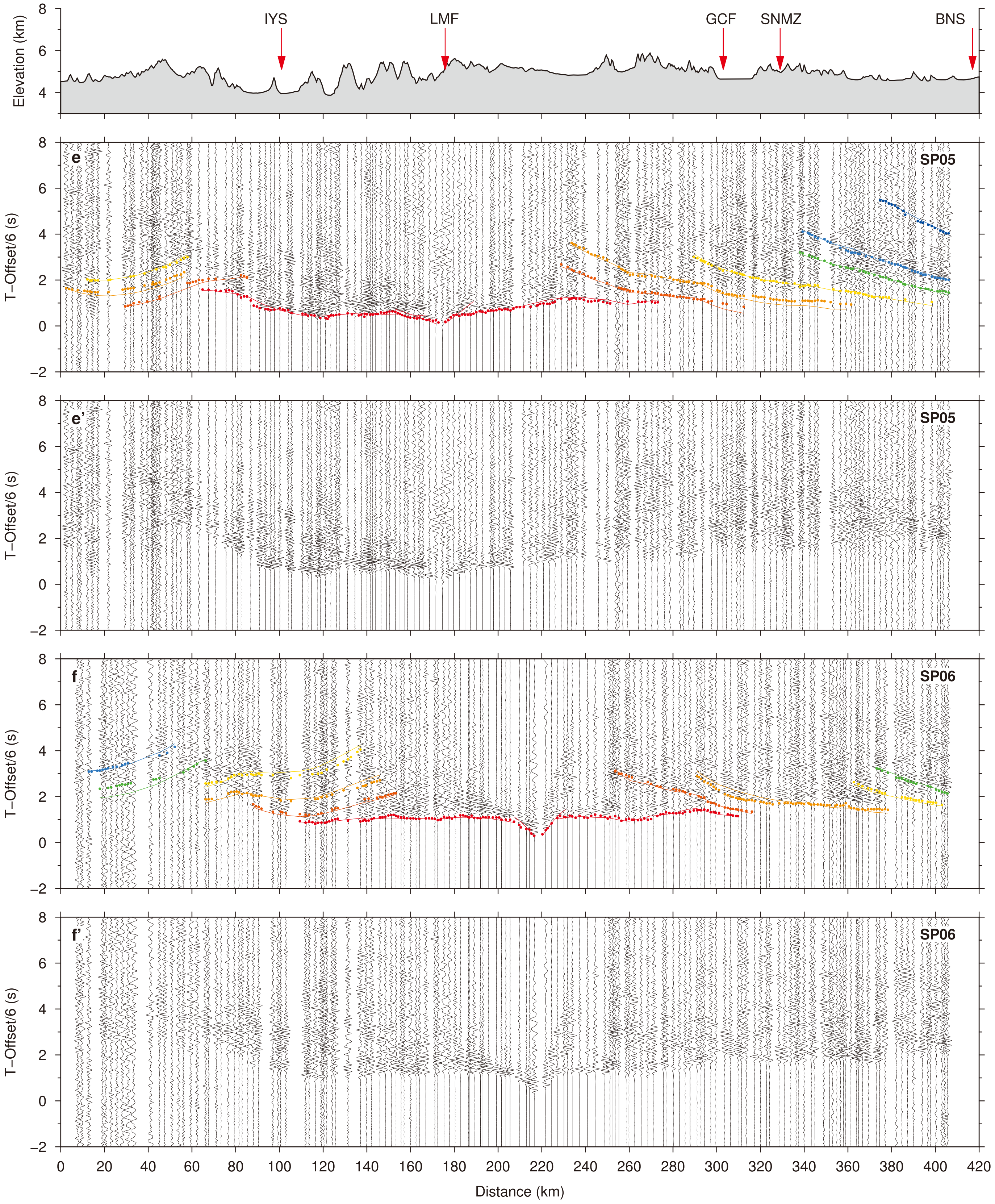

Supplementary Figure 2. Seismic sections for the ten shots (continued).

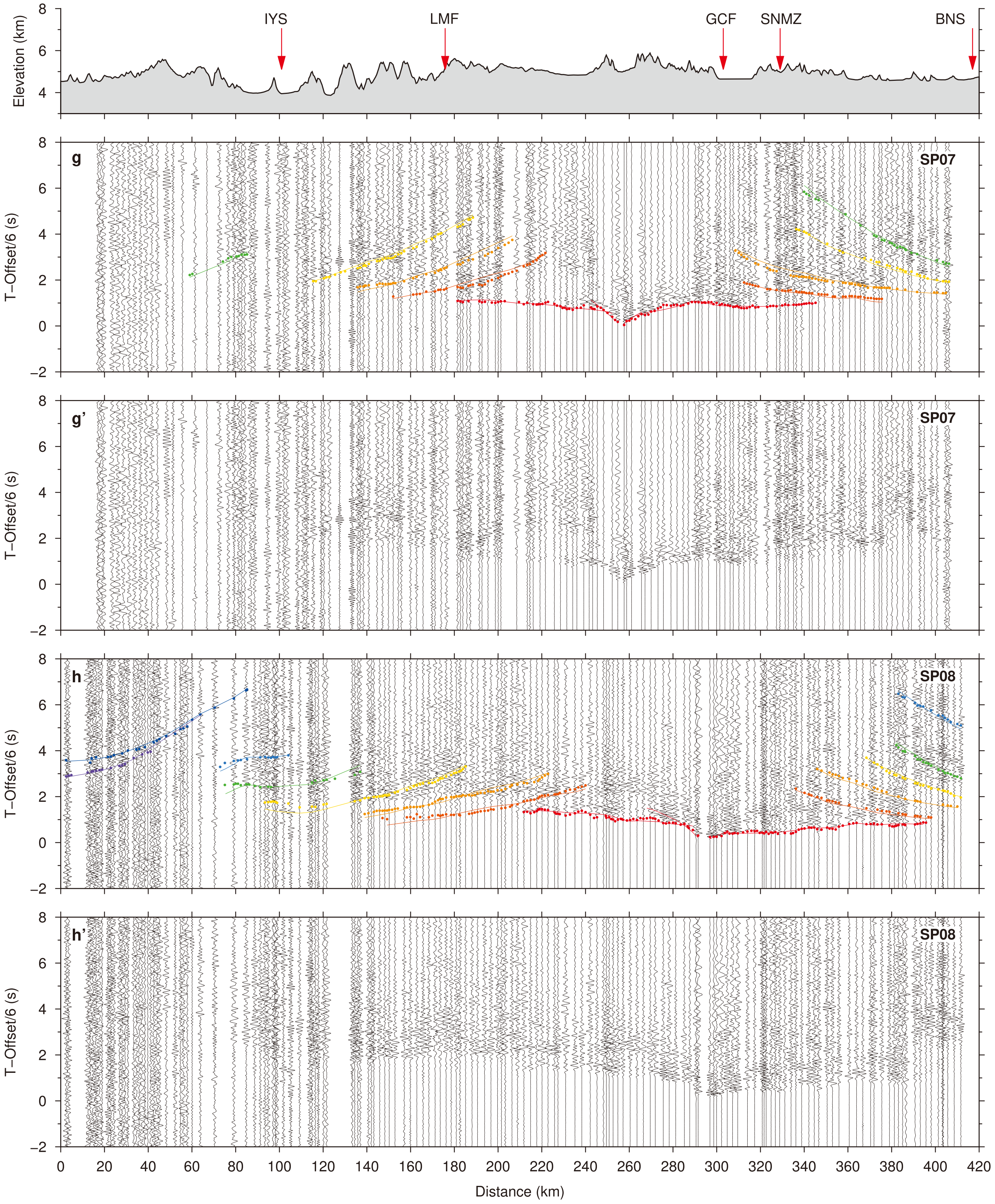

Supplementary Figure 2. Seismic sections for the ten shots (continued).

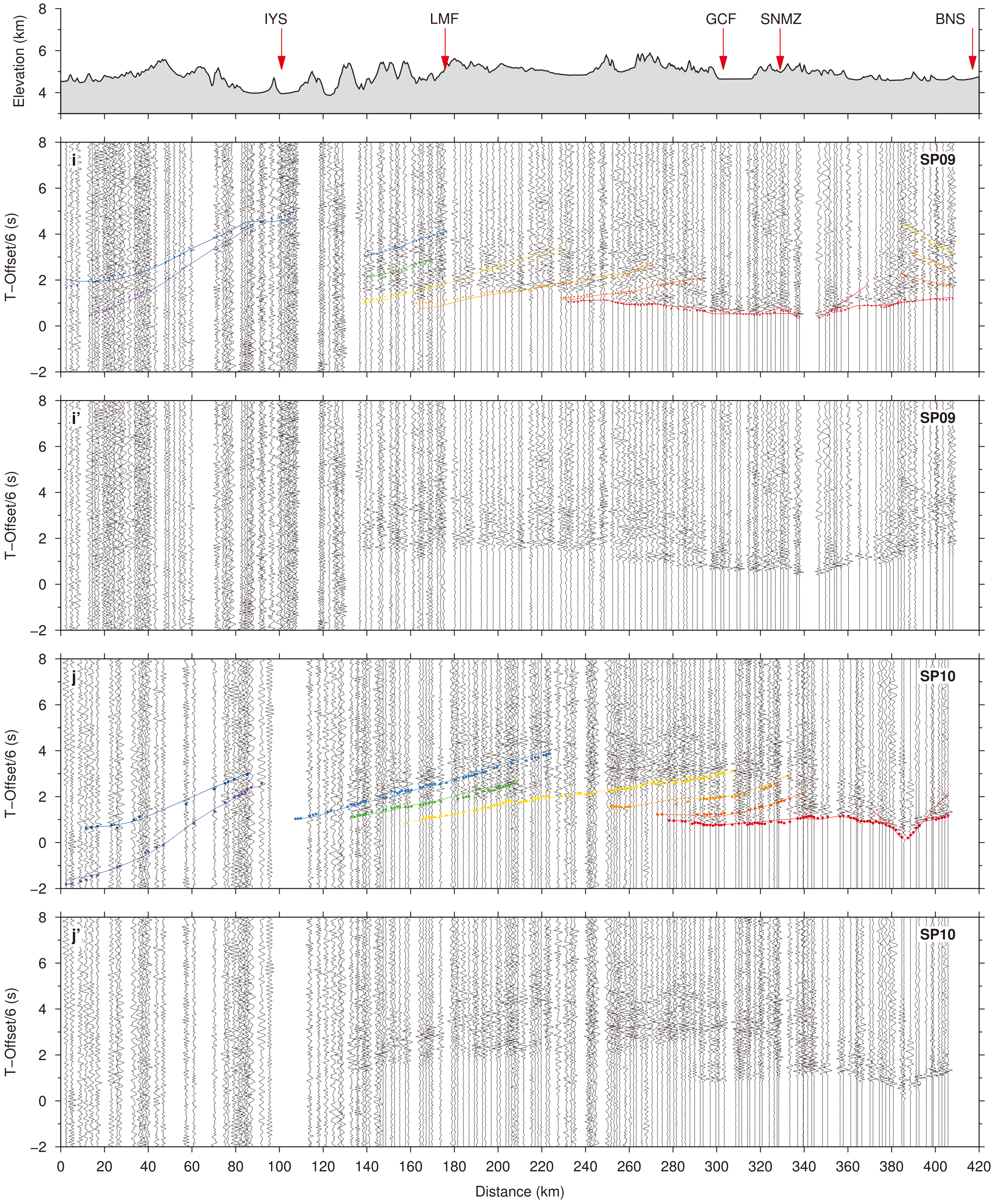

Supplementary Figure 2. Seismic sections for the ten shots (continued).

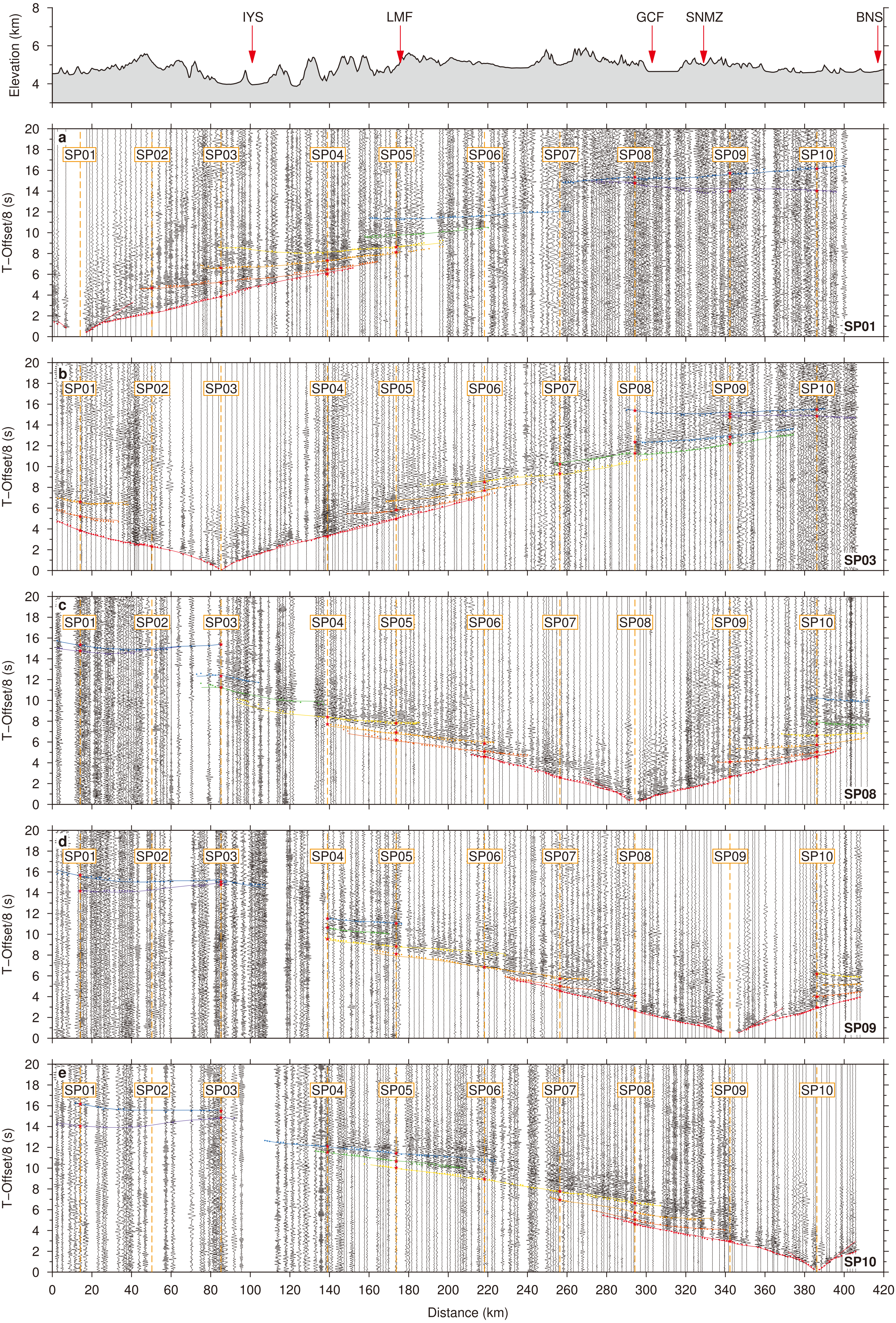

Elevation (km)
IYS
LMF
GCF
SNMZ
BNS
a
b
c
d
e
SP01
SP02
SP03
SP04
SP05
SP06
SP07
SP08
SP09
SP10
T–Offset/8 (s)
Distance (km)

Supplementary Figure 3. Seismic sections with reduction velocity 8 km/s to enhance the PmP reflection from Moho and the Pn refraction from below Moho for the most relevant shot point SP01, SP03, SP08, Sp09, and SP10. Our traveltime picks are shown by coloured dots. Locations of shot points along the profile are shown and reciprocal traveltimes are marked by red stars.

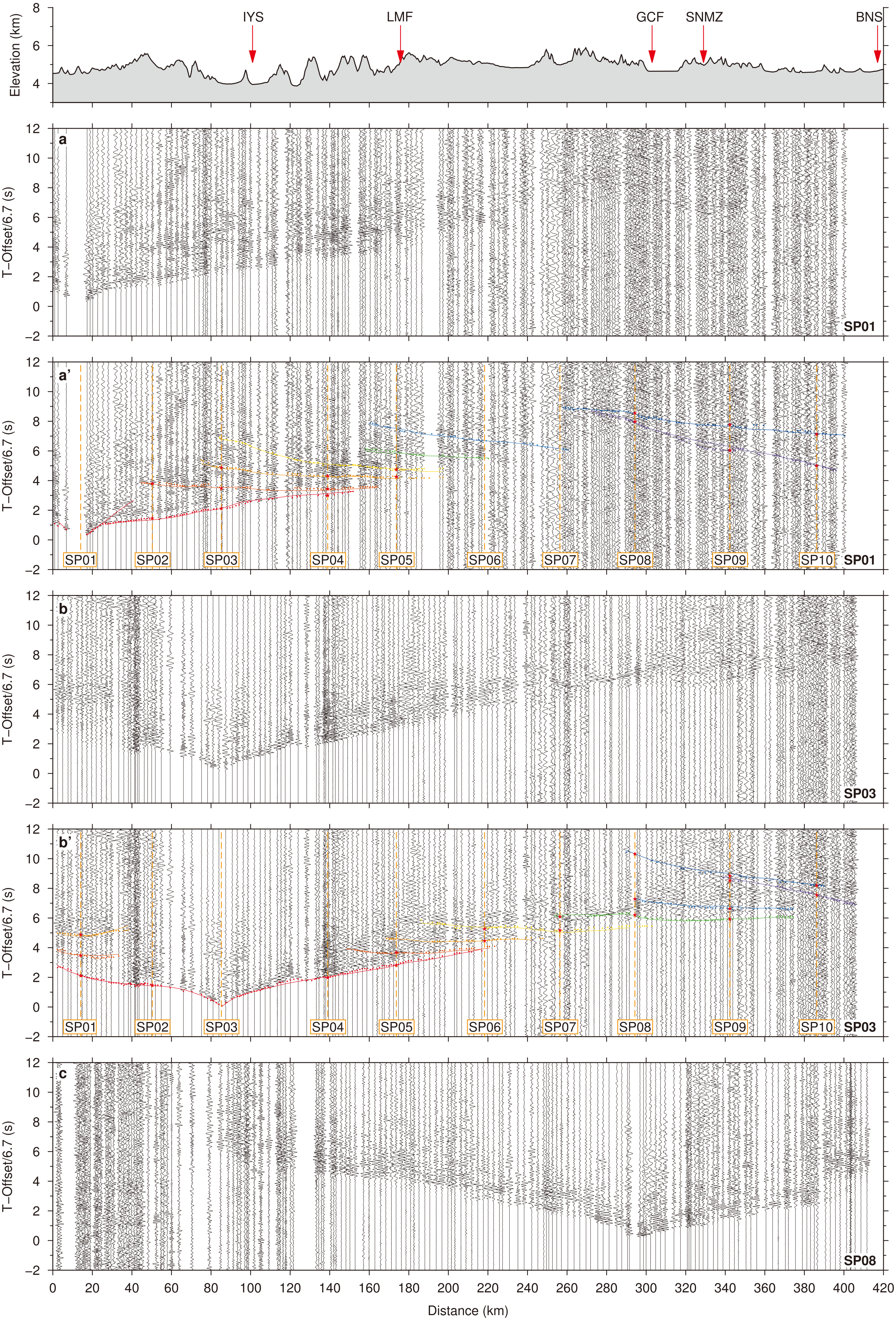

IYS
LMF
GCF
SNMZ
BNS
Elevation (km)
T−Offset/6.7 (s)
a
a’
b
b’
c
SP01
SP02
SP03
SP04
SP05
SP06
SP07
SP08
SP09
SP10
Distance (km)

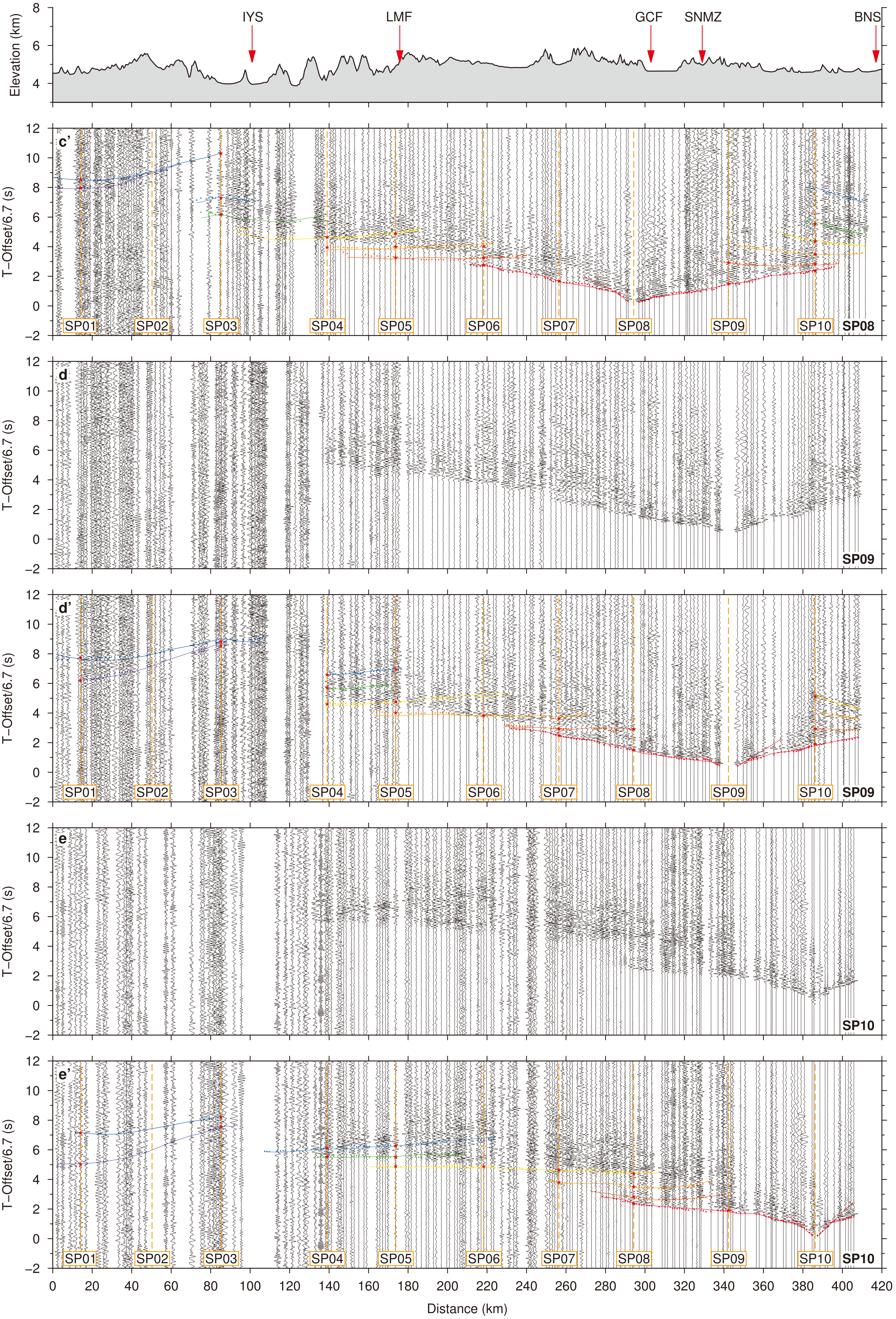

Elevation (km)
IYS
LMF
GCF
SNMZ
BNS
T−Offset/6.7 (s)
c’
SP01
SP02
SP03
SP04
SP05
SP06
SP07
SP08
SP09
SP10
SP08
d
SP09
d’
SP09
e
SP10
e’
SP10
Distance (km)

Supplementary Figure 4. Seismic sections for shot points 1, 3, 8, 9, and10. For each shot point, the upper panel shows the section, and the lower panels shows the seismic section with the picks (dots) and the traveltimes (solid lines) calculated for the final velocity model. Topographic profile with major tectonic features is inserted on top of each set of seismic sections. The travel time is reduced by a velocity of 6.7 km/s to facilitate interpretation of the far field PmP reflections from the Moho. Please notice that the PmP reflections are stronger in the down-dip (southern) direction (SP08 and SP10) than in the up-dip (northern direction (SP01 and SP03). Application of the reciprocity principle therefore facilitated secure identification of the PmP arrival for SP01 and SP03. Abbreviations: IYS: Indus–Yarlung suture, LMF: Luobadui–Milashan fault, GCF: Gyaring Co fault, SNMZ: Shiquan River–Nam Tso Mélange Zone, BNS: Bangong- Nujiang suture.

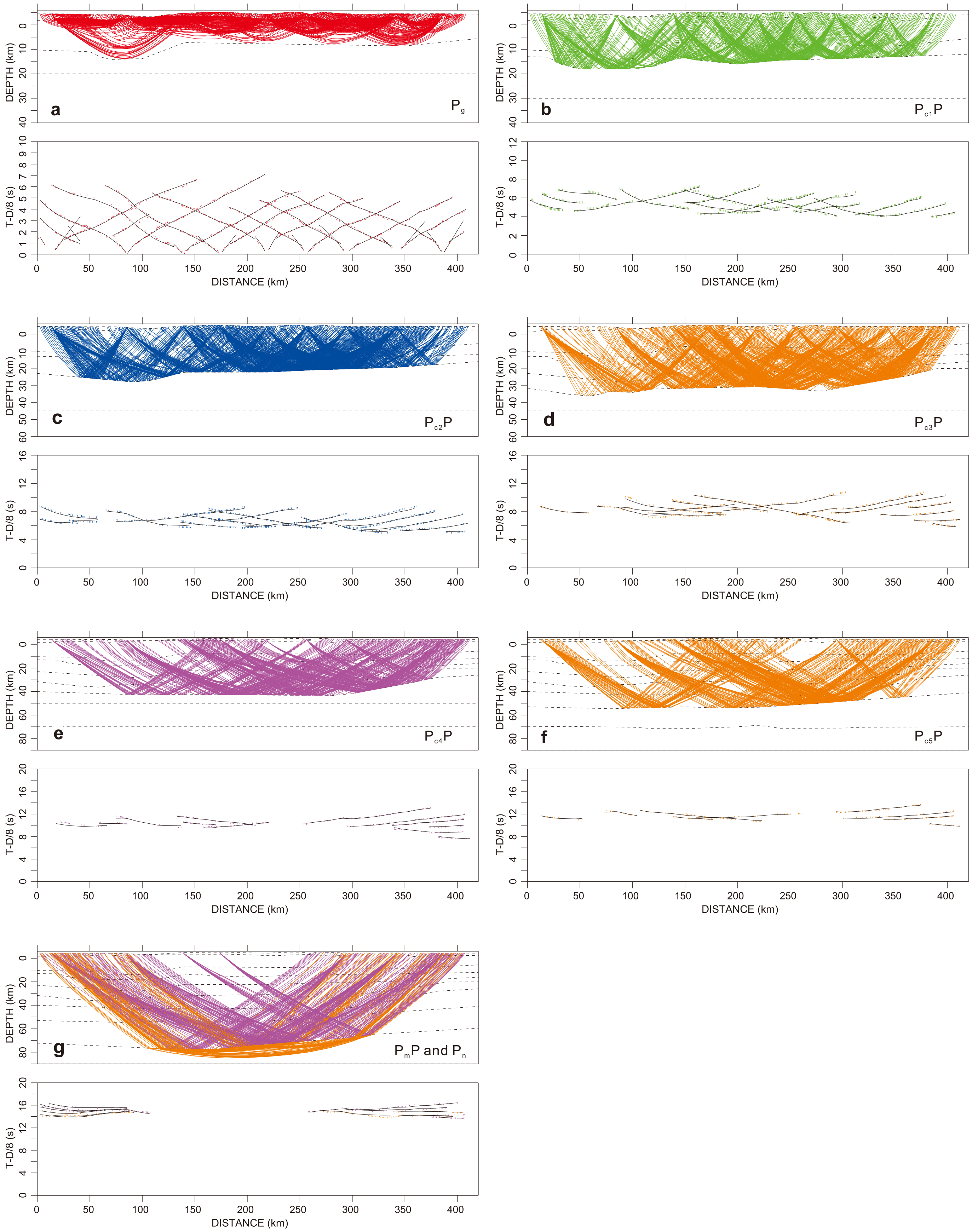

Supplementary Figure 5. Ray tracing results for different seismic phases. Ray tracing results calculated by the RAYINVR program. The upper panels show the rays and the bottom panels show the picks (vertical bars of length corresponding to uncertainty) and the calculated times (solid lines) corresponding to the rays shown in the upper panels.

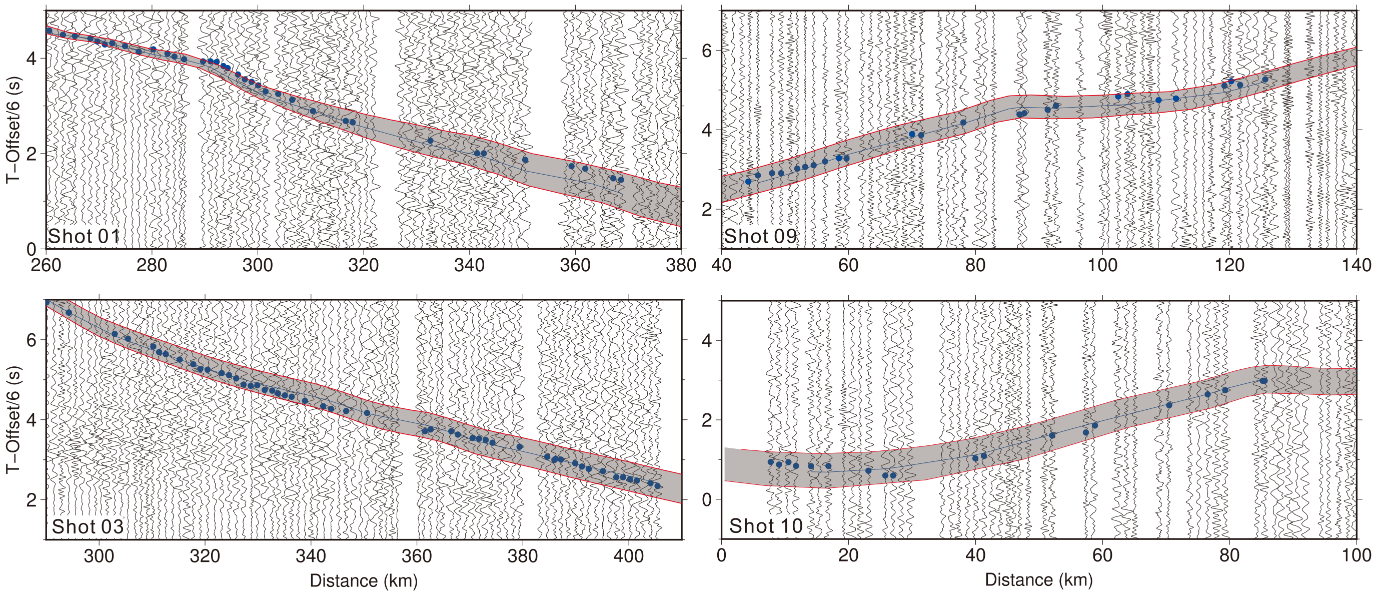

Supplementary Figure 6. Test of lower crustal velocity. The calculated arrival times of the P_mP phases with different velocities in the lower crust. The shaded zones between the top and bottom red lines show the arrival times between the velocity of 6.5 km/s (upper red lines) and 6.8 km/s (bottom red lines). The dots show the picked P_mP arrival times.

i. Inversion for Moho depth with lower crustal velocity fixed at 7.0 km/s

a

Iteration	Phase	Total number of picks	Number of picks included in inversion	t_{RMS}(ms)	χ^2
0	P_mP,P_n	279	273	574	33.113
1	P_mP,P_n	279	265	255	6.516
2	P_mP,P_n	279	267	230	5.322
3	P_mP,P_n	279	274	229	5.253
4	P_mP,P_n	279	258	230	5.318
5	P_mP,P_n	279	259	224	5.040
**6**	**P_mP,P_n**	**279**	**264**	**220**	**4.873**
7	P_mP,P_n	279	249	267	7.139
8	P_mP,P_n	279	254	257	6.610
9	P_mP,P_n	279	240	289	8.359
**Preferred Model**	**P_mP,P_n**	**279**	**279**	**99**	**0.986**

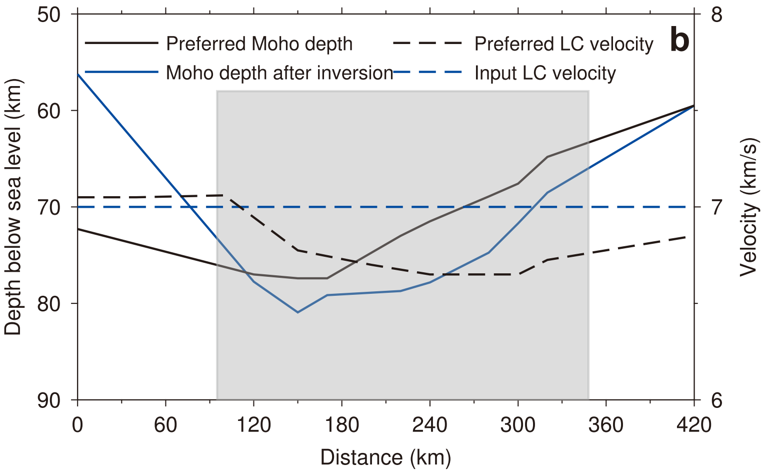

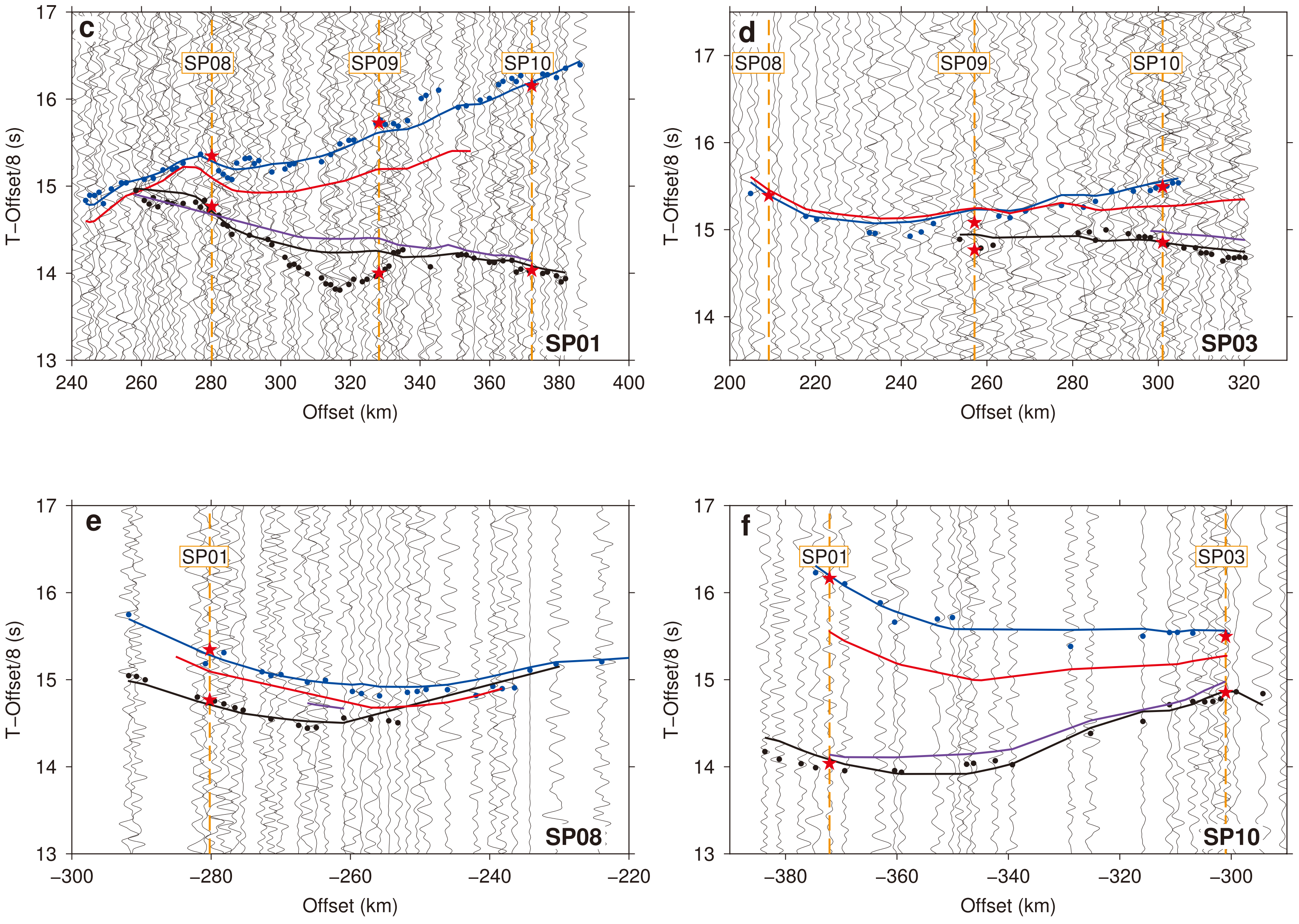

ii. Inversion for Moho depth and LC velocity for input LC velocity = 7.0 km/s

a

Iteration	Phase	Total number of picks	Number of picks included in inversion	t_{RMS}(ms)	χ^2
0	P_mP, P_n	279	273	574	33.113
1	P_mP, P_n	279	265	108	1.177
2	P_mP, P_n	279	268	114	1.303
3	P_mP, P_n	279	274	104	1.087
4	P_mP, P_n	279	258	82	0.678
5	P_mP, P_n	279	254	218	4.770
**Preferred Model**	**P_mP, P_n**	**279**	**279**	**99**	**0.986**

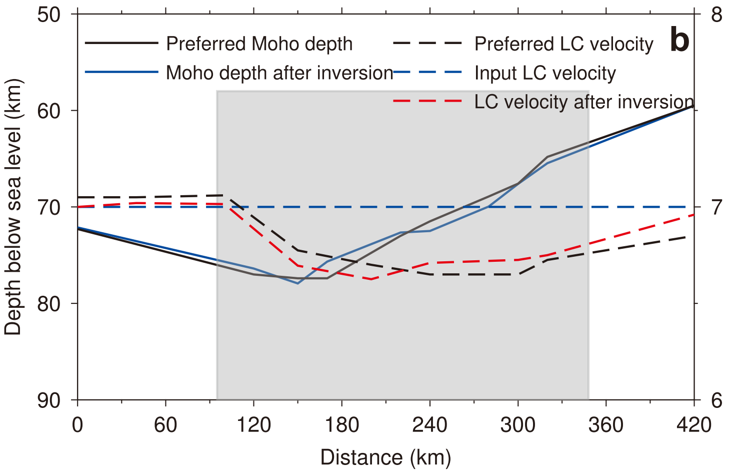

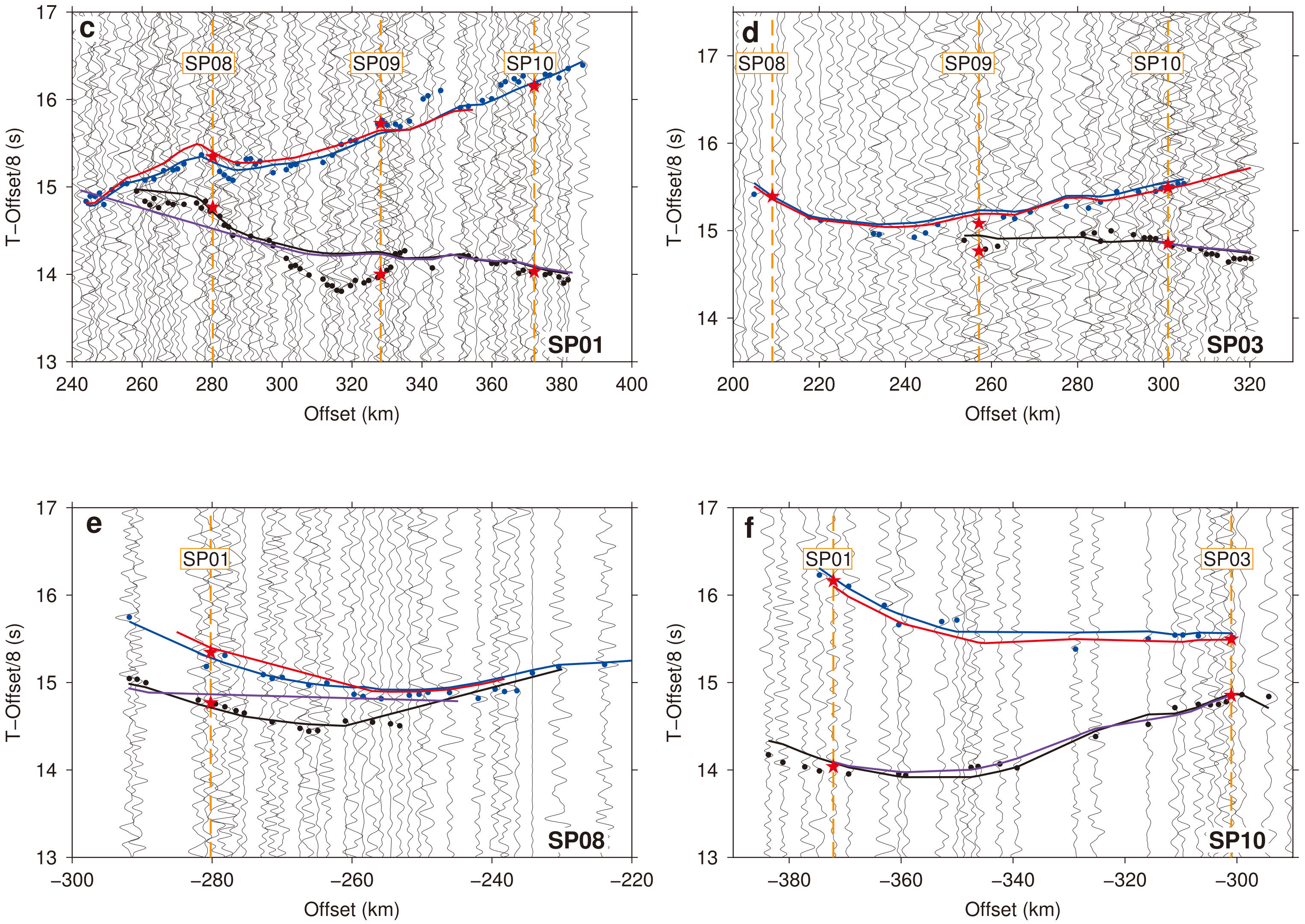

iii. Inversion for LC velocity for Moho fixed 5 km deeper than in preferred model

a

Iteration	Phase	Total number of picks	Number of picks included in inversion	t_{RMS}(ms)	χ^2
0	P_mP,P_n	279	269	755	57.258
**1**	**P_mP,P_n**	**279**	**260**	**225**	**5.060**
2	P_mP,P_n	279	257	243	5.951
3	P_mP,P_n	279	256	251	6.304
4	P_mP,P_n	279	254	316	10.023
5	P_mP,P_n	279	259	248	6.185
6	P_mP,P_n	279	260	264	6.974
**Preferred Model**	**P_mP,P_n**	**279**	**279**	**99**	**0.986**

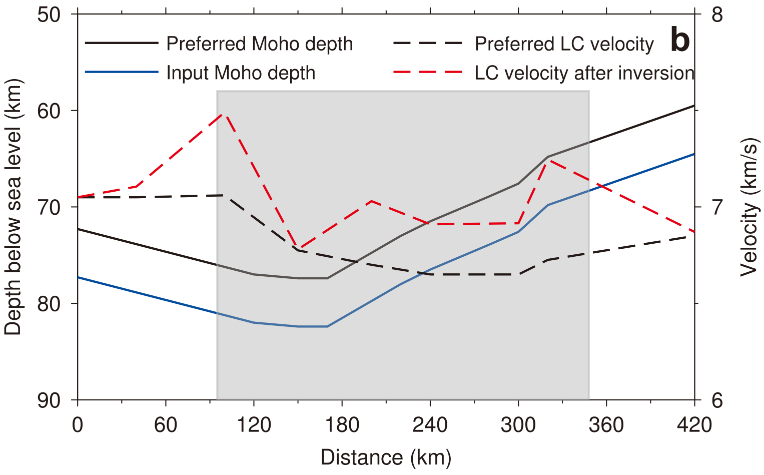

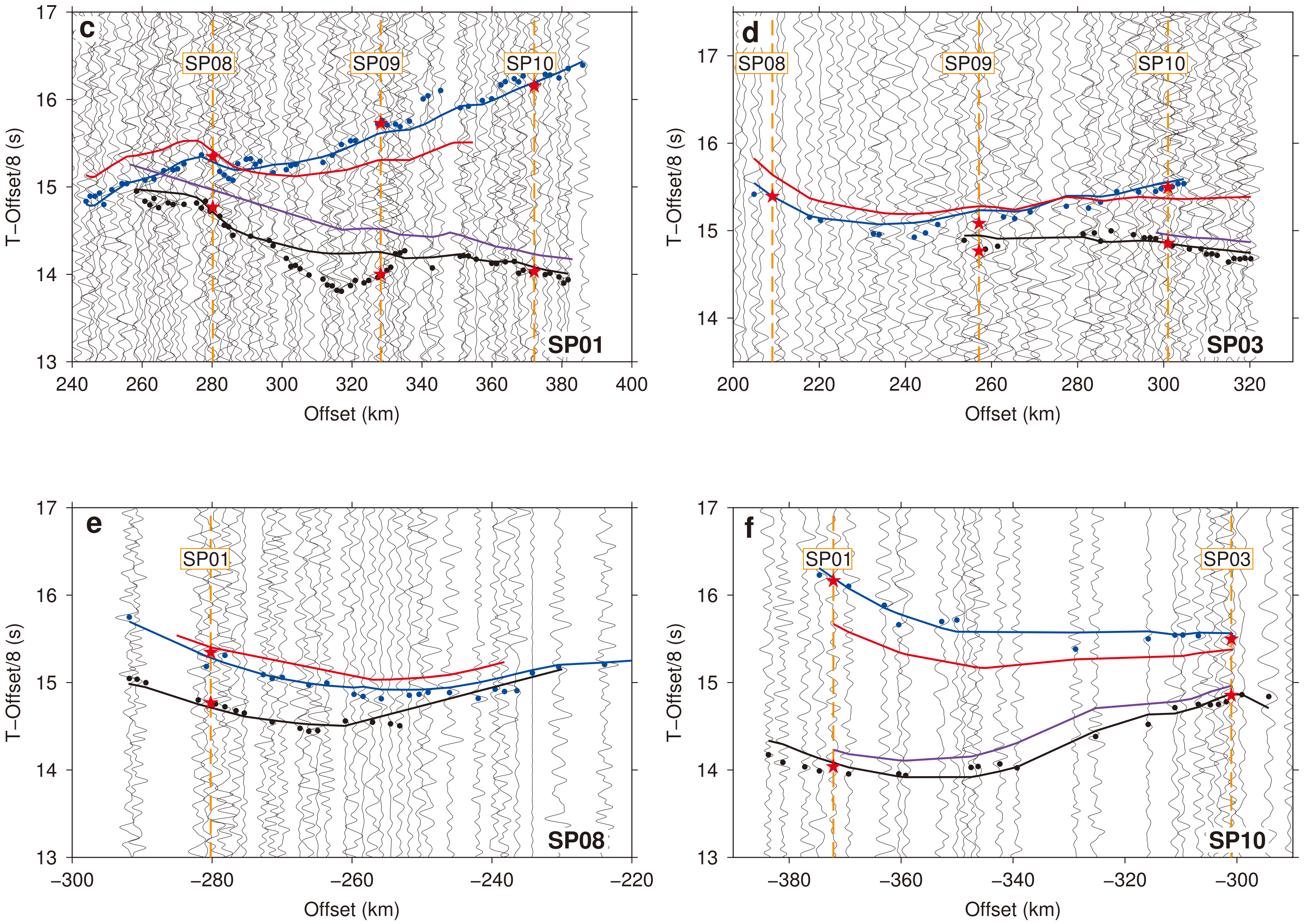

iv. Inversion for Moho depth and LC velocity for initial Moho depth 5 km deeper than in preferred model

a

Iteration	Phase	Total number of picks	Number of picks included in inversion	t_{RMS}(ms)	χ^2
0	P_mP,P_n	279	269	755	57.258
1	P_mP,P_n	279	201	124	1.547
2	P_mP,P_n	279	255	109	1.198
3	P_mP,P_n	279	220	79	0.633
4	P_mP,P_n	279	214	84	0.709
5	P_mP,P_n	279	235	164	2.712
**6**	**P_mP,P_n**	**279**	**259**	**115**	**1.339**
7	P_mP,P_n	279	220	94	0.883
**Preferred Model**	**P_mP,P_n**	**279**	**279**	**99**	**0.986**

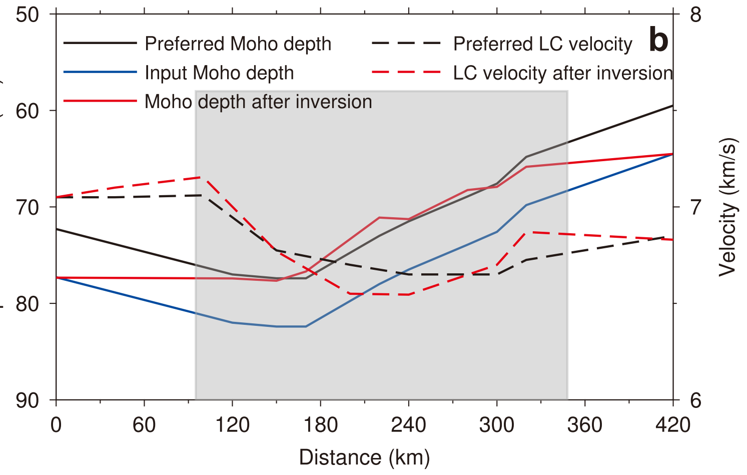

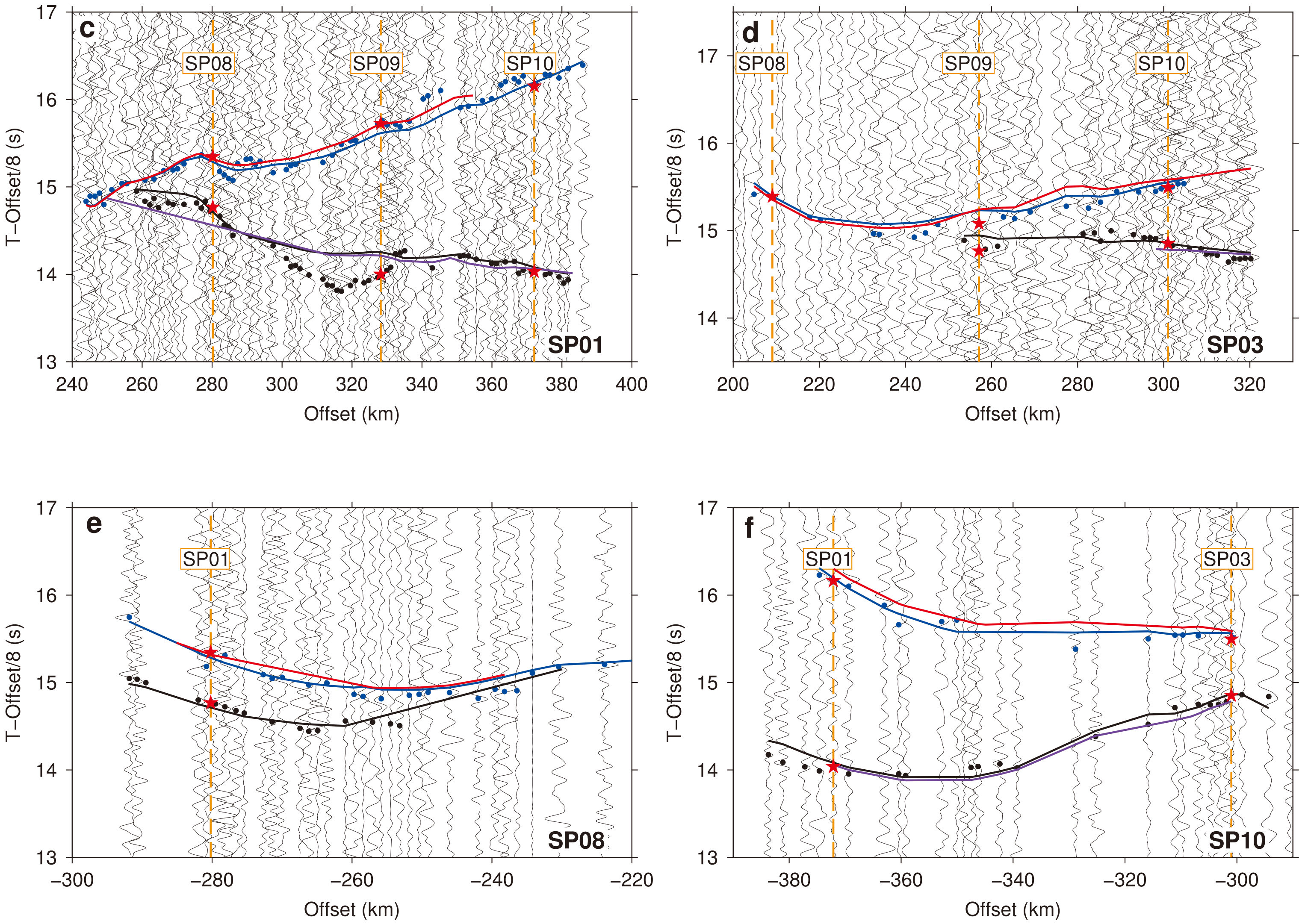

Supplementary Figure 7. Robustness test of lower crustal velocity and Moho depth. i. Test if lower crustal velocity may be fixed at 7.0 km/s by least-squares inversion for the best Moho depth; test shows that no model can explain observations within uncertainty of traveltime picks as the best obtainable model has a mis-fit of 220 ms; and P_n arrivals cannot be matched for SP01 and SP10. **ii.** Inversion for lower crustal velocity and Moho depth for initial model with 7.0 km/s; resulting model is similar to our preferred model. **iii.** Test if Moho depth may be fixed 5 km deeper than in preferred model by leastsquares inversion for the lower crustal velocity; test shows that no model can explain observations within uncertainty of traveltime picks as the best obtainable model has a mis-fit of 225 ms. **iv.** Inversion for lower crustal velocity and Moho depth for initial model with Moho 5 km deeper than in our preferred model; resulting model is similar to our preferred model. In each panel: **a.** Inversion quality for the P_mP and P_n phases for each iteration, including total number of picks; resulting No. of picks explained; rms traveltime misfit (ms) where uncertainty is estimated to 100 ms; and the χ_2 which ideally should be around 1 for best fit model, selected best-fit final model is marked by red text. **b.** Variation of Moho depth and lower crustal velocity along the profile for preferred model, initial model and selected final model after inversion. **c-f.** Zoomed seismic sections for SP01, Sp03, SP08 and Sp10 with traveltime picks and calculated traveltimes for preferred model (P_mP: blue, P_n: black) and for resulting model after inversion (P_mP: red, P_n: purple). Location of reversed shot points are marked and reciprocal traveltimes are marked by red stars.

Supplementary Table 1. Shot Parameters for the data acquisition

Shot points Number	Latitude(°)	Longitude(°)	Altitude(m)	Time(UTC)y:d:h:m:s	TNT charge (kg)
SP01	28.3354	88.6076	4869	2016:281:15:20:31.380	4000
SP02	28.6984	88.5707	4961	2016:281:15:32:22.150	4000
SP03	29.0166	88.4986	4390	2016:281:21:42:05.710	2000
SP04	29.4911	88.3290	4695	2016:270:00:21:42.385	2000
SP05	29.8087	88.3838	5386	2016:269:22:24:08.480	2000
SP06	30.2126	88.5072	5249	2016:268:15:34:05.335	2000
SP07	30.5543	88.6155	4811	2016:268:15:17:22.440	2000
SP08	30.8821	88.8201	4871	2016:262:14:11:04.145	2000
SP09	31.3252	88.6564	4778	2016:253:00:34:59.440	4000
SP10	31.7230	88.5143	4594	2016:252:14:39:29.300	4000

Supplementary Table 2. Statistical parameters of the seismic modeling for different picks.

Phase Number	Total number of picks	The number of picks for modeling	t_{RMS}(ms)	χ^2
P_g	1038	1029	80	2.574
$P_{C1}P$	533	531	100	0.995
$P_{C2}P$	659	653	135	1.827
$P_{C3}P$	544	544	117	1.361
$P_{C4}P$	339	339	115	1.317
$P_{C5}P$	273	273	88	0.782
P_mP,P_n	279	279	99	0.986
Total	3665	3648	104	1.645
